# Optimal noise level for coding with tightly balanced networks of spiking neurons in the presence of transmission delays

**DOI:** 10.1371/journal.pcbi.1010593

**Published:** 2022-10-17

**Authors:** Jonathan Timcheck, Jonathan Kadmon, Kwabena Boahen, Surya Ganguli

**Affiliations:** 1 Department of Physics, Stanford University, Stanford, California, United States of America; 2 Department of Applied Physics, Stanford University, Stanford, California, United States of America; 3 Department of Bioengineering, Stanford University, Stanford, California, United States of America; Queen’s University, CANADA

## Abstract

Neural circuits consist of many noisy, slow components, with individual neurons subject to ion channel noise, axonal propagation delays, and unreliable and slow synaptic transmission. This raises a fundamental question: how can reliable computation emerge from such unreliable components? A classic strategy is to simply average over a population of *N* weakly-coupled neurons to achieve errors that scale as $1/\sqrt{N}$. But more interestingly, recent work has introduced networks of leaky integrate-and-fire (LIF) neurons that achieve coding errors that scale *superclassically* as 1/*N* by combining the principles of predictive coding and fast and tight inhibitory-excitatory balance. However, spike transmission delays preclude such fast inhibition, and computational studies have observed that such delays can cause pathological synchronization that in turn destroys superclassical coding performance. Intriguingly, it has also been observed in simulations that noise can actually *improve* coding performance, and that there exists some optimal level of noise that minimizes coding error. However, we lack a quantitative theory that describes this fascinating interplay between delays, noise and neural coding performance in spiking networks. In this work, we elucidate the mechanisms underpinning this beneficial role of noise by deriving *analytical* expressions for coding error as a function of spike propagation delay and noise levels in predictive coding tight-balance networks of LIF neurons. Furthermore, we compute the minimal coding error and the associated optimal noise level, finding that they grow as power-laws with the delay. Our analysis reveals quantitatively how optimal levels of noise can rescue neural coding performance in spiking neural networks with delays by preventing the build up of pathological synchrony without overwhelming the overall spiking dynamics. This analysis can serve as a foundation for the further study of precise computation in the presence of noise and delays in efficient spiking neural circuits.

## Author summary

Remarkably, the brain can perform precise computations, despite being composed of noisy neurons with slow, unreliable synaptic connectivity. To understand how this is possible, we can imagine the classic strategy where neurons are grouped into weakly-coupled subpopulations, creating redundancy to achieve high precision. But interestingly, recent work proposed a tight-balance neural network that instead uses fast, strong connectivity between neurons to achieve much higher precision with the same number of neurons. This efficiency is attractive, but notably, signals take time to propagate in the brain. Such propagation delays alone can lead to pathological synchronization. Intriguingly, while noise commonly degrades the performance of a computational system, it has been observed in simulations that noise can help mitigate synchronization and in fact rescue performance in tight-balance networks. In this work, we develop a theory that quantifies the simultaneous effects of delays and noise in tight-balance networks, and allows us to compute the optimal noise level as a function of delay, yielding conceptual insights into how noise can counteract delay induced synchronization to preserve precise computation in efficient neural networks.

## Introduction

The brain is capable of precise, reliable computation—for example, a professional violinist generates fine motor commands to reproduce a given pitch, or an impressionist produces speech patterns to mimic a celebrity’s voice. Yet, the underlying computational substrates in the brain—neurons, synapses, and axonal transmission—are noisy, unreliable, and slow [1, 2]. This paradox begs a fundamental question in neuroscience: how do neural networks facilitate precise computation with imprecise computational primitives [3]? And moreover, in light of evolutionary forces favoring energy-efficiency [4, 5], how is this precise computation facilitated *efficiently*?

The simplest setting to study the precision of computation is that of coding [6]—with what fidelity can, say, a dynamical signal *x*(*t*) be encoded in a network and read back out as an estimate $\hat{x}\left(t\right)$? The classic strategy is to read out $\hat{x}\left(t\right)$ as an average over *N* redundant neurons; this results in a readout error that scales as $1/\sqrt{N}$ as long as single neuron noise is not strongly coupled across the population [1]. However, a recent predictive coding framework [7] introduced a formulation for a tightly-balanced network of spiking leaky-integrate-and-fire (LIF) neurons with readout error that scales *superclassically* as 1/*N*. In predictive coding, only the unpredicted difference $\hat{x}\left(t\right)-x\left(t\right)$ is encoded and passed to downstream processing, saving a great deal of information compared to directly encoding *x*(*t*); signatures of predictive coding have been observed in sensory areas of the brain [8]. The predictive coding framework [7] combines this principle with that of a strong, fast inhibitory feedback, known as tight balance, so that each neural spike corrects the error $\hat{x}\left(t\right)-x\left(t\right)$ when it reaches a threshold and rapidly inhibits the other neurons to prevent overcorrections—this results in a highly efficient code, in which no spike is wasted. Moreover, despite each spike’s dedicated purpose, the framework is robust to the death of individual neurons and reproduces the highly irregular spiking activity observed in cortex [9].

Critically, however, axonal and synaptic transmission introduce a delay that renders the rapid inhibition in the framework [7] problematic, reducing the fidelity of the code [10–12]. Namely, if an inhibitory signal arrives late, other neurons may spuriously spike, producing overcorrections in the error and wasted spikes. However, intriguingly, adding noise to the neural membrane potentials introduces a beneficial variation, spreading out the times at which neurons will spike next so that the delayed inhibition has sufficient time to propagate before spurious spikes occur, and rescues the coding fidelity. Notably, too little noise does not provide a sufficient spread, and too much noise destroys overall fidelity. Thus an optimal noise level exists.

Importantly, several simulation studies have observed the beneficial role of noise in the predictive coding framework [7], regardless of the specific neural model or noise modality. For example, [12] studies a soft-threshold neural model with transmission delays, [11] studies an LIF model with membrane noise, transmission delays, and synaptic delays, and [10] studies conductance-based Hodgkin-Huxley neural dynamics with finite time-scale synapses. Indeed, it is a general phenomenon in the predictive coding framework that a group of neurons compete to correct the same error, and delays preclude timely inhibition, resulting in pathological synchrony known as the hipster effect [13]; noise helps diversify the dynamics, assuaging the effect. While observations from simulations are insightful, however, we lack a quantitative understanding of this fascinating interplay between the delay and the level of noise. How does coding fidelity depend on the length of the delay and the level of noise? And given a delay, what is the highest achievable fidelity and the associated optimal level of noise? Indeed, the simple, efficient spiking network of the predictive coding framework presents a foundational scenario in which to expand the study of stochastic facilitation [14].

We address these fundamental questions by going beyond simulations to derive analytical expressions for coding fidelity as a function of noise level and small delays in tightly balanced networks of LIF neurons. Previous work [15] derived similar expressions for non-spiking “rate” neurons by adapting the predictive coding framework [7] to non-spiking neurons. Our work takes a step closer toward understanding efficient coding in biological neural networks by explicitly including the spiking nature of neural communication in the brain. Indeed, when considering efficiency, spikes are important because action potentials account for a large portion of the brain’s energy expenditure [16] and provide a form of digital communication, which may allow the brain to tap into the efficiency associated with a hybrid analog-digital computing system [17, 18]. Moreover, experiments have shown that spike-timing conveys information in several brain regions [19–21]. Thus, we hope that our analytical insights here provide a foundation for further investigation into the interplay of noise and delays in efficient cortical circuits.

## Models

### Efficient coding with a network of leaky integrate and fire neurons

We ask the question, how well can a spiking network encode a continuous, time-varying input signal in the presence of noise and transmission delays? To operationalize this question, we start with three assumptions: (1) the output signal is linearly decoded from a densely-connected population of spiking neurons, (2) the network minimizes the mean-squared error between its output and the input signal, and (3) for brevity, we assume the input is 1-dimensional, though our results can be extended to multi-dimensional signals. Thus we consider a scalar input signal *x*(*t*), and the network’s scalar output $\hat{x}\left(t\right)$—the network’s estimate for *x*(*t*). The network itself is a densely-connected recurrent circuit of *N* leaky integrate and fire (LIF) neurons. The activity of the *i*’th neuron (where *i* = 1, …, *N*) is described by the spike train, $o_{i}\left(t\right)=\sum_{k}\;\delta \left(t-t_{i}^{k}\right)$, where *δ*(⋅) is the Dirac *δ*-function representing a single spike, and $t_{i}^{k}$ is the time of the *k*’th spike of the *i*’th neuron. To convert this discrete spiking activity into a smoother output signal, the spike trains *o*_*i*_(*t*) are first passed through a linear filter to yield the instantaneous firing rates *r*_*i*_(*t*), which obey

$$\begin{array}{c} \tau {\dot{r}}_{i}\left(t\right)=-r_{i}\left(t\right)+\tau o_{i}\left(t\right), \end{array}$$ (1)

where the dot (˙) represents derivative with respect to time, and *τ* is the time-constant of the filter. And second, these firing rates *r*_*i*_(*t*) are linearly summed to yield the network’s estimate $\hat{x}\left(t\right)$:

$$\begin{array}{c} \hat{x}\left(t\right)≔\frac{1}{N}\sum_{i=1}^{N}w_{i}r_{i}\left(t\right), \end{array}$$ (2)

where the $w_{i}\in R$ are the weights of the linear decoder.

The network’s objective is to achieve $\hat{x}\left(t\right)\approx x\left(t\right)$ by minimizing the mean-squared-error $\varepsilon^{2}={\langle {\left(x\left(t\right)-\hat{x}\left(t\right)\right)}^{2}\rangle }_{t}$, where the angular brackets 〈⋅〉_*t*_ denote average over time. In order to simplify our study of the mean-squared-error *ε*^2^, we choose to work with inputs *x*(*t*) that vary slowly compared to the spiking network’s timescale *τ*. With this choice, we can treat the input *x*(*t*) = *x* as effectively constant, and thus *ε*^2^ can be written as $\varepsilon^{2}={\left({\langle \hat{x}\left(t\right)\rangle }_{t}-x\right)}^{2}+{\langle {\left(\delta \hat{x}\left(t\right)\right)}^{2}\rangle }_{t}$, where we have substituted in $\delta \hat{x}\left(t\right)≔\hat{x}\left(t\right)-{\langle \hat{x}\left(t^{\prime }\right)\rangle }_{t^{\prime }}$ and used the fact that ${\langle \delta \hat{x}\left(t\right)\rangle }_{t}=0$. Importantly, we see that *ε*^2^ can be divided into two contributions: a contribution from the bias ${\left({\langle \hat{x}\left(t\right)\rangle }_{t}-x\right)}^{2}$ and a contribution from the variance ${\langle {\left(\delta \hat{x}\left(t\right)\right)}^{2}\rangle }_{t}$. Since the bias—here, a constant—could be deterministically removed [15], we focus on computing the contribution from the variance, which is simply the square of the standard deviation of the readout,

$$\begin{array}{c} \sigma_{readout}=\sqrt{{\left\langle {\left(\hat{x}\left(t\right)-{\left\langle \hat{x}\left(t^{\prime }\right)\right\rangle }_{t^{\prime }}\right)}^{2}\right\rangle }_{t}}. \end{array}$$ (3)

We henceforth refer to *σ*_*readout*_ as the readout error—an inverse measure for coding fidelity.

The dynamics of the *N* densely-connected LIF neurons are given by the equation

$$\begin{array}{c} \begin{array}{cc} & \tau {\dot{V}}_{i}\left(t\right)=-\lambda_{V}V_{i}\left(t\right)+I_{i}\left(t\right)-\tau J_{ii}o_{i}\left(t\right)-\tau \sum_{j\neq i}\;J_{ij}o_{j}(t-\Delta )+\sqrt{\tau }\sigma \eta_{i}\left(t\right)\text{,}\;\text{and}\;\\ & \text{and}\;\text{neuron}\;i\;\text{emits}\;\text{a}\;\text{spike}\;\text{when}\;V_{i}>T. \end{array} \end{array}$$ (4)

where *V*_*i*_(*t*) is the membrane potential of the *i*’th neuron, $T=\frac{1}{2}$ is the firing threshold (in the absence of noise, delay, and with small leak, one will find that this choice of threshold will make the average membrane potential approximately zero when the estimation error is zero [7]), λ_*V*_ controls the strength of the leak, *I*_*i*_(*t*) is the input current, *J*_*ii*_ implements the neural self-reset, *J*_*ij*_ implements dense connectivity, Δ is the spike propagation delay, and the *η*_*i*_(*t*) are independent unit-Gaussian noise with *σ* controlling the membrane noise level.

Now the critical question is, how do we choose the input *I*_*i*_(*t*) and recurrent connectivity *J*_*ij*_ so that the noisy, delayed, discontinuous, and nonlinear dynamics of Eq 4 result in minimal readout error *σ*_*readout*_? We start with the predictive coding framework proposed in [7] that specifies these network properties given fixed readout weights {*w*_*i*_: 1 ≤ *i* ≤ *N*}. The framework does so by minimizing the mean-squared-error in a setting in which there are no delays (Δ = 0), and noise is negligible. The resulting network [7] has

$$\begin{array}{c} J_{ij}=w_{i}w_{j} \end{array}$$ (5)

$$\begin{array}{c} I_{i}\left(t\right)=Nw_{i}x\left(t\right). \end{array}$$ (6)

This network exhibits tight balance: the *O*(*N*) excitatory input currents *I*_*i*_(*t*) are matched by the *O*(*N*) inhibitory terms involving *J*_*ij*_ in Eq 4. (The weights *w*_*i*_ and the input *x*(*t*) are *O*(1), and each neuron, on average, spikes at an *O*(1) rate, hence a total *O*(*N*) inhibition when summing across the population.) Tight balance, i.e., balance of *O*(*N*), can be contrasted with balance of $O(\sqrt{N})$, known as classical balance [22], or balance of $O(<\sqrt{N})$, representing loose or no balance. Importantly, tight balance facilitates the superclassical *O*(1/*N*) scaling of the readout error in [7]—as was shown in [15]—and thus we specialize in this work to tight balance. And for simplicity, we specialize to uniform readout weights, *w*_*i*_ = 1 ∀*i*, which corresponds to a population of neurons with the same tuning curve. In the following, we study how the nonzero delay Δ and non-negligible noise level *σ* impact the readout error *σ*_*readout*_.

### Soft-threshold model

In addition to the LIF model, we introduce a soft-threshold model to study the effects of noise and delays in a simpler setting. In the soft-threshold model, the membrane potentials obey

$$\begin{array}{c} \begin{array}{cc} & \tau {\dot{V}}_{i}\left(t\right)=I_{i}\left(t\right)-\tau J_{ii}o_{i}\left(t\right)-\tau \sum_{j\neq i}J_{ij}o_{j}(t-\Delta )\text{,}\;\\ & \text{and}\;\text{neuron}\;\text{i}\;\text{emits}\;\text{spikes}\;\text{with}\;\text{probability}\;\text{rate}\;\rho \left(V_{i}\right)=\{\begin{array}{cc} \rho , & \;V_{i}>T\\ 0, & \;V_{i}\leq T \end{array}. \end{array} \end{array}$$ (7)

The soft-threshold model is also known as the escape-rate model [23], and has been used in prior work on the predictive coding framework [12] and in fitting neural spike train recordings to Generalized Linear Models (GLMs) [24].

Notably, the probabilistic firing of the neurons introduces variation in spike-timing. This is similar to how in the LIF model (Eq 4) the noise term $\sqrt{\tau }\sigma \eta_{i}\left(t\right)$ accumulates over time in the membrane potentials, which also results in variation in spike-timing. Intuitively, the standard deviation, 1/*ρ*, of the exponentially-distributed spike-times under the probabilistic firing rate *ρ* corresponds to an effective noise level, which we can tune by adjusting *ρ*. However, note that in contrast to the LIF model, which requires the leak term λ_*V*_ *V*_*i*_(*t*) to bound accumulated variability from the noise term $\sqrt{\tau }\sigma \eta_{i}\left(t\right)$, we will see in the next section that probabilistic firing introduces naturally-bounded spike-time variability. Thus, the leak term λ_*V*_ *V*_*i*_(*t*) is not necessary in the soft-threshold model, and so we do not include it in Eq 7 for simplicity.

Importantly, for the *ρ* → ∞, zero delay (Δ = 0) limit, the soft-threshold model becomes equivalent to the original formulation of the predictive coding framework with hard threshold [7]. The LIF model also becomes equivalent to [7] in the zero noise (*σ* = 0), zero delay (Δ = 0) limit. Thus, both models serve as good starting points for analyzing the predictive coding framework with small delays and small noise, as we will see below. We analyze the soft-threshold model in addition to the more complex LIF model because it offers simpler derivations, but yields similar conclusions as the more complex LIF model.

## Results

### Overall behavior of efficient coding spiking models

**Fig 1:**
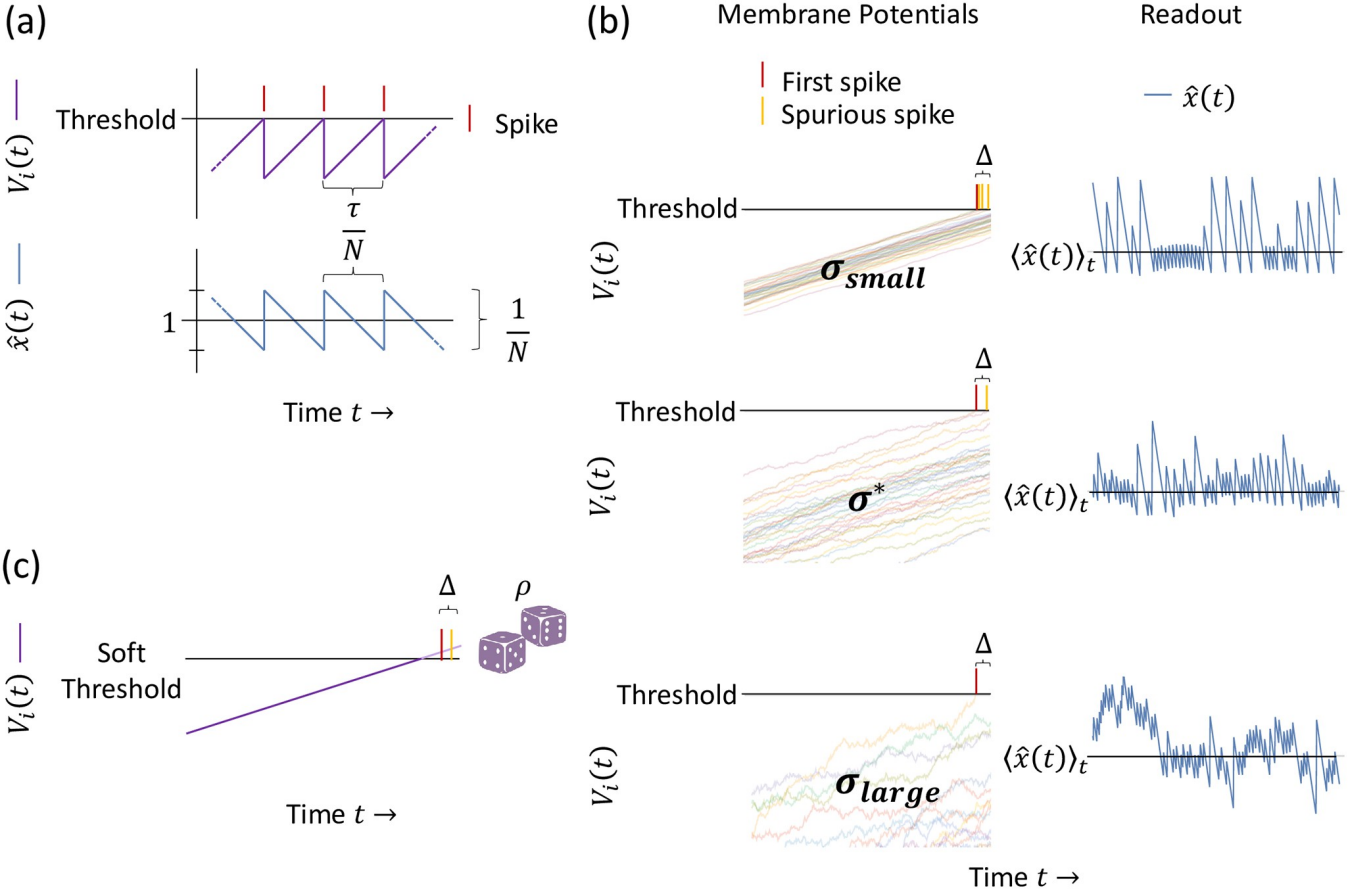
Tight-balance spiking network dynamics and readout. (a) Nominal dynamics. When there are no spike propagation delays and zero noise, the membrane potentials (purple) follow the same trajectory in time. When the population reaches threshold, one neuron’s spike (red) instantaneously inhibits all the neurons, preventing further spikes. This produces perfectly regular spikes, like clockwork with an approximate period of $\frac{\tau }{N}$. Each spike contributes $\frac{1}{N}$ to the readout, creating a tight, zig-zag approximation $\hat{x}\left(t\right)$ (blue) for the encoded signal, *x*(*t*) = 1 in this case. (b) The effect of delays and noise. When delays are present and noise is added to the membrane potentials (left, multicolor), two effects appear that decrease the fidelity of the readout $\hat{x}\left(t\right)$ (right, blue): variation in spike-timing and synchronous spurious spikes. The noise on the membrane potentials (*σ*) creates variations in the time it takes membrane potentials to reach threshold—a deviation from the perfectly regular spikes in (a). And after the first neuron in the population crosses threshold and spikes (red), there is a delay Δ until the other neurons receive inhibition, and thus some extra neurons may spike—spurious synchronous spikes (yellow). Given a fixed delay, too little noise *σ*_*small*_ does not spread the membrane potentials enough to prevent a large number of spurious spikes, and too much noise *σ*_*large*_ destroys the fidelity of the code altogether. An optimal noise level exists, σ*. (c) Soft-threshold model. In the soft-threshold model, neurons spike probabilistically once their membrane potentials surpass threshold, with a spiking probability rate *ρ*. As *ρ* is varied (not illustrated), one finds a relationship analogous to the noise level trade-off for the LIF model shown in (b): too small *ρ* creates large variations in spike times, and too large *ρ* creates many spurious spikes during the delay. Thus, an optimal ρ* exists.

To understand the nominal dynamics of the LIF and soft-threshold models, let us consider encoding the constant input signal *x*(*t*) = 1, and first consider the dynamics of the LIF model with a large number of neurons *N*. The input current *I*_*i*_(*t*) (Eq 6) becomes *I*_*i*_(*t*) = *N* with our choice of decoding weights *w*_*i*_ = 1, and the connectivity strengths *J*_*ij*_ become *J*_*ij*_ = 1 ∀*i*, *j* (Eq 5). When there are no spike transmission delays (Δ = 0) and no noise (*σ* = 0), the dynamics of every membrane potential *V*_*i*_(*t*) are identical (Eq 4): a spike from any neuron inhibits all membrane potentials equally, instantaneously, and simultaneously; and any differences in the membrane potentials’ initial conditions *V*_*i*_(0) are forgotten on the time-scale *τ*/λ_*V*_ due to the leak term −λ_*V*_ *V*_*i*_(*t*). We are interested in continuously operating networks, so let us consider the network dynamics after a long time *t* ≫ *τ*/λ_*V*_, in which the initial conditions are indeed forgotten. In this case, the membrane potentials *V*_*i*_(*t*) are traveling together toward threshold, driven by the input current *I*_*i*_(*t*) = *N* (Fig 1a, top). When a membrane potential reaches threshold, the neuron fires a spike, which immediately self-resets the membrane potential by 1 via the −*τJ*_*ii*_*o*_*i*_(*t*) term and decrements all other membrane potentials by 1 through the −*τ* ∑_*j*≠*i*_ *J*_*ij*_*o*_*j*_(*t*) term (Eq 4). (Note that here we have assumed that some infinitesimal variation in the membrane potentials persists, e.g., an infinitesimal remnant from the forgotten initial conditions; thus, when the membrane potentials approach threshold, a single membrane potential hits threshold and spikes an instant before the rest of the membrane potentials, allowing sufficient, i.e., infinitesimal, time for the *instantaneous* inhibition to prevent additional neurons from spiking.) The membrane potentials then continue to be driven by the input current *I*_*i*_(*t*) = *N*, and it takes a time of approximately *τ*/*N* for a membrane potential to reach threshold again, where we have assumed that the leak term −λ_*V*_ *V*_*i*_(*t*) is small relative to the *O*(*N*) driving current because the membrane potentials themselves are *V*_*i*_(*t*) = *O*(1). When a membrane potential reaches threshold after time of approximately *τ*/*N*, it fires a spike, and this process repeats: the network produces spikes like clockwork, with an approximate period of *τ*/*N*.

Given this network spiking pattern, we can understand the corresponding readout trajectory $\hat{x}\left(t\right)$ by recalling that the readout $\hat{x}\left(t\right)$ is a sum of instantaneous firing-rates *r*_*i*_(*t*) uniformly weighted by $\frac{1}{N}$ (Fig 1a, bottom). The mean readout ${\langle \hat{x}\left(t\right)\rangle }_{t}=1$ because the time constant of the instantaneous firing rates *r*_*i*_(*t*) is *τ* (Eq 1), *τ* × 1/(*τ*/*N*) = *N* spikes occur during a time *τ*, and the firing rates are weighted by $\frac{1}{N}$ in the decoder (*N* spikes $\times \frac{1}{N}=1$). This mean matches the desired signal, *x*(*t*) = 1. Furthermore, the readout trajectory $\hat{x}\left(t\right)$ forms a zig-zag around the mean value because the readout $\hat{x}\left(t\right)$ simply exponentially decays with time constant *τ* between spike-times, which is approximately linear because the time between spikes *τ*/*N* is small compared to *τ* (since *N* is large). And the zig-zag is tight; it has magnitude *O*(1/*N*), because each spike contributes $\frac{1}{N}$ to the readout. Thus we see here the superclassical *O*(1/*N*) scaling of the readout error, because each individual spike is precisely timed to optimally correct the deviations in $\hat{x}\left(t\right)$ from *x*(*t*) = 1.

Next, let us consider the addition of noise *σ* > 0 and spike propagation delay Δ > 0 (Fig 1b). Intuitively, the integration of the independent noise terms $\sqrt{\tau }\sigma \eta_{i}\left(t\right)$ spread out the membrane potentials *V*_*i*_(*t*), and thus they no longer share exactly the same trajectory when traveling toward threshold and instead travel in a continuously-fluctuating packet of some finite width. Importantly, both the packet width and the variance in the time to first spike increase with the noise level *σ*. Now, as the packet travels toward threshold, a top neuron in the packet eventually reaches threshold and spikes. This spike instantly self-resets the firing neuron through the −*τJ*_*ii*_*o*_*i*_(*t*) term, but the inhibition arrives a time Δ later to the other membrane potentials through the −*τ* ∑_*j*≠*i*_ *J*_*ij*_*o*_*j*_(*t* − Δ) term. Importantly, during the delay time Δ, all membrane potentials continue to be driven by the strong input current *I*_*i*_(*t*) = *N*, and so there is a possibility that additional membrane potentials reach threshold before they receive the inhibition from the first neuron’s spike. As these neurons hit threshold, they produce extra, spurious spikes that create undesirably large deviations in the readout $\hat{x}\left(t\right)$, leading to high readout error. Thus for a fixed delay Δ, we see a trade-off in the noise level: for small noise *σ*_*small*_, the membrane potentials travel in a tight packet, and thus there are likely many membrane potentials crossing threshold during the delay Δ resulting in many spurious spikes and a large readout error. And for large noise *σ*_*large*_, the membrane potentials are more spread out, reducing the number of spurious spikes during the delay Δ, but at the cost of introducing a large deviation in the time-to-spike for the first-spiking, top neuron in the packet. This implies that there exists some intermediate optimal noise level σ* that balances these effects to minimize the readout error. Below we will analytically compute the readout error *σ*_*readout*_ as a function of the noise level *σ* and the delay Δ and calculate this optimal noise level σ* and the associated minimal readout error $\sigma_{readout}^{*}$.

Importantly, the soft-threshold model exhibits the same trade-off (Fig 1c). For small probabilistic firing rate *ρ*, the number of spurious spikes during the delay Δ is small, but the standard deviation in the time-to-spike of a single neuron is large: 1/*ρ*. And for large *ρ*, the number of spurious spikes is large, but the standard deviation in time-to-spike is small. Thus for some fixed delay Δ, an optimal ρ* (or equivalently, an optimal noise level 1/ρ*) exists that minimizes the readout error *σ*_*readout*_ in the soft-threshold model.

In the following subsections, we quantitatively elucidate this trade-off between minimizing spurious spikes and minimizing spike-timing variability, and we calculate *σ*_*readout*_ as a function of noise and delay for the soft-threshold model and the LIF model in turn, for networks with a large number of neurons *N*. We begin with the simpler soft-threshold model, as it will allow us to derive an exact expression for *σ*_*readout*_ in the limit of small delays and noise. Then, we will analyze small noise and delays in the LIF model, and ultimately derive an approximate upper-bound for *σ*_*readout*_ and see that both models exhibit the same behavior. We corroborate our analytic results with simulations, whose details are provided in Section B Simulation details in S1 Appendix.

### Analysis of noise, delays, and coding error in the soft-threshold model

In this section, we calculate the contributions of spike-time variability and spurious spikes to the readout error *σ*_*readout*_ for the soft-threshold model. We provide concise derivations here, and detailed derivations in S1 Appendix. To begin, we consider the simple scenario in which the membrane potentials *V*_*i*_(*t*) all start with initial condition *V*_*i*_(0) = 0, as would be the case when there is no external input, and if there were an additional leak term to ensure all membrane potentials decay to 0. Then, we consider turning on the dynamics, Eq 7. The input current *I*_*i*_(*t*) = *N* drives the membrane potentials toward threshold together, and all membrane potentials reach threshold simultaneously. At this point, the population begins emitting spikes probabilistically with rate *Nρ*, as there are *N* membrane potentials superthreshold individually firing with probability rate *ρ*.

#### Statistics of spike-time variability

**Fig 2:**
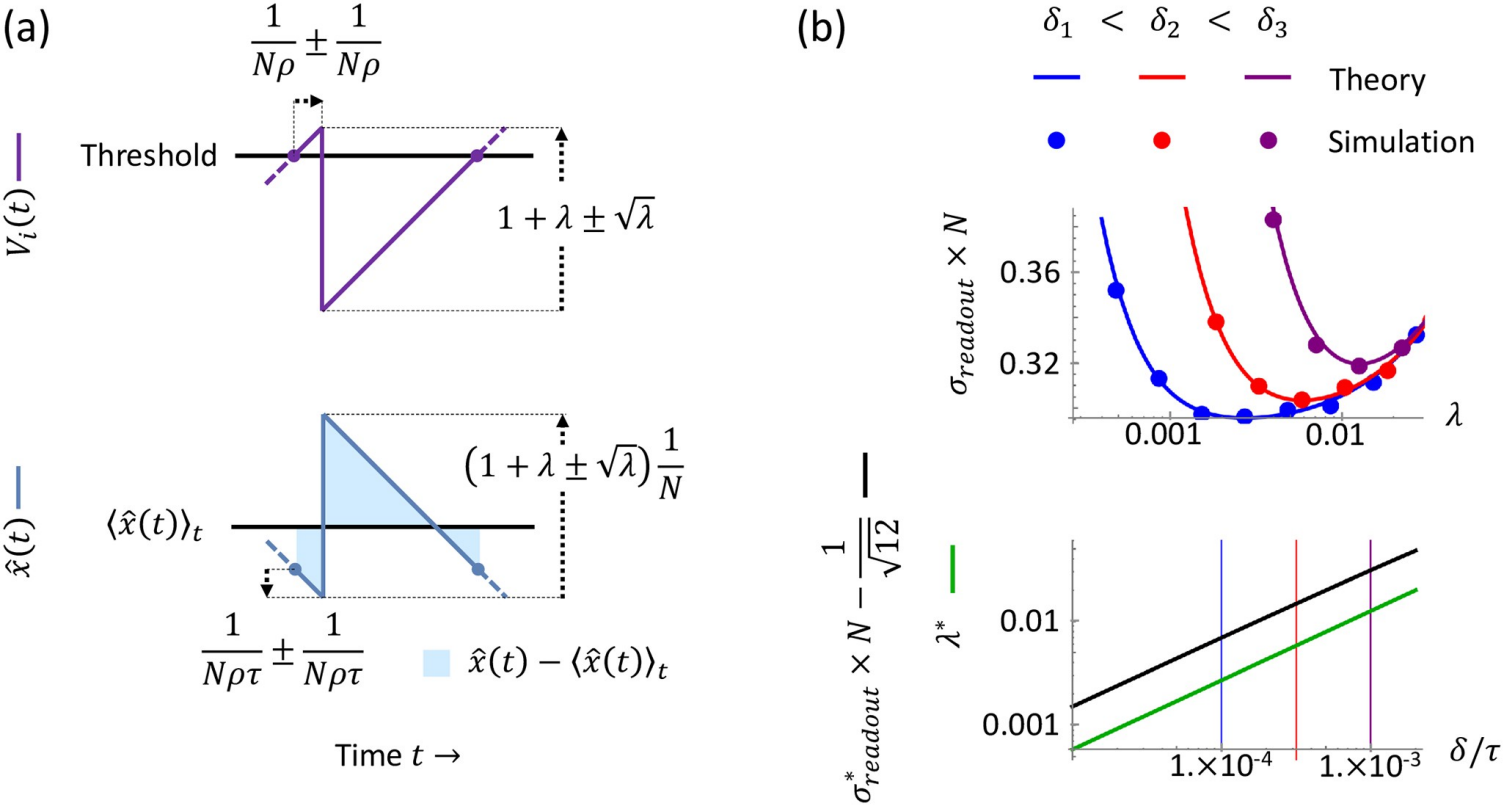
Soft-threshold readout error. (a) The soft-threshold and the delay create variations in the membrane potential dynamics *V*_*i*_(*t*), which in turn create variations in the readout $\hat{x}\left(t\right)$. When the membrane potentials (top, purple) surpass threshold, the neurons spike probabilistically, and the first-spike time is an exponential random variable with standard deviation $\frac{1}{N\rho }$. After a first spike, the number of spurious spikes that occur during the delay is a Poisson random variable, with standard deviation $\sqrt{\lambda }$, and each spike inhibits the membrane potentials *V*_*i*_(*t*) by 1 through recurrent connectivity (see the first paragraph of Results for recurrent connectivity). These variations in spike-timing and spurious spikes carry through to the readout $\hat{x}\left(t\right)$ (bottom, blue). Note that since the network input is constant, the readout encoding this input should produce a constant output as closely as possible; however, these variations instead increase the deviation (light blue shaded) from the mean readout ${\langle \hat{x}\left(t\right)\rangle }_{t}$ (black horizontal line). (b) Readout error as a function of the mean number of spurious spikes λ and the delay *δ*. Top: for three different values of delay (blue, red, purple), λ is varied in computer simulations (N = 32, dots) and Eq 10 (solid curves), revealing both the U-shaped dependence of the readout error *σ*_*readout*_ and an excellent match between theory and experiment. Bottom: the optimal readout error $\sigma_{readout}^{*}$ (black) and the associated optimal λ* increase as a function of delay *δ* according to Eqs 12 and 11.

After some time above threshold, the population eventually emits a spike. The variation in this first-spike time (where time is measured relative to when the population had crossed threshold) is a departure from the optimal clockwork one-spike-every-*τ*/*N* spiking pattern for a network with zero delay and zero noise as described above, and thus this first-spike time variability increases the readout error *σ*_*readout*_. To quantify the increase, we therefore wish to describe the statistics of this first-spike time. Now, since the population is emitting spikes probabilistically with a constant rate (*Nρ*), the time it takes until the first spike occurs is an exponentially-distributed random variable. For an exponential distribution, the mean and standard deviation are given by the reciprocal of the rate, thus here the first-spike time has mean $\frac{1}{N\rho }$ and standard deviation $\frac{1}{N\rho }$ (Fig 2a, top). And naturally, this first-spike time variability also creates fluctuations in the readout, with standard deviation $\frac{1}{N\rho \tau }$, which arises from the standard deviation of the first-spike time $\frac{1}{N\rho }$, multiplied by the magnitude of the $-\frac{1}{\tau }$ slope of the approximately linear decay of the readout (Fig 2a, bottom).

#### Statistics of spurious spikes induced by delays

After the first spike occurs, one membrane potential is instantly reset, and the other *N* − 1 membrane potentials continue to spike probabilistically during the spike propagation delay Δ. Hence, spurious spikes may occur, and we would like to describe their statistics to quantify how they increase the readout error *σ*_*readout*_. To this end, the mean number of spurious spikes, λ, is the time Δ multiplied by the total spiking probability rate of the population, which is (*N* − 1)*ρ* ≈ *Nρ*; this yields

$$\begin{array}{c} \lambda =\Delta N\rho . \end{array}$$ (8)

And here, for ease of analysis, we assume the delay Δ is much smaller than the *O*(1/*N*) network interspike interval (see the second paragraph of Results for the *O*(1/*N*) interspike interval). With this assumption, differences in the *N* membrane potentials due to delayed inhibition vanish before the membrane potentials impinge upon threshold together again; this guarantees the simple scenario that we are considering here, where *N* neurons always reach threshold together (see Section A.2 Readout error for the soft-threshold model in S1 Appendix for details). Thus we introduce the parameter *δ* by the definition

$$\begin{array}{c} \Delta ≔\frac{\delta }{N} \end{array}$$ (9)

where we assume *δ* ≪ *τ* so that the delay Δ is much less than the network inter-spike interval. Note that with these definitions we have λ = *δρ*. And since we are interested in high-performing networks, i.e. small readout error *σ*_*readout*_, we focus on the limit where λ ≪ 1, where there are few undesirable spurious spikes. In this limit, the number of spurious spikes is simply Poisson-distributed with mean λ and standard deviation $\sqrt{\lambda }$. Importantly, the variability in the number of spurious spikes creates variability in the next time the population reaches threshold together because each spike decrements the membrane potentials, thus the input current *I*_*i*_(*t*) = *N* takes a variable amount of time to drive the membrane potentials to threshold again (Fig 2a, top). And the fluctuation in the readout due to spurious spikes has standard deviation $\sqrt{\lambda }/N$, as each spike contributes $\frac{1}{N}$ to the readout (Fig 2a, bottom).

#### Averaging across time to calculate the readout error

To calculate *σ*_*readout*_, we recognize that the time-average of the squared deviation of the readout in Eq 3 is by definition an integral of the squared deviation over a long time interval, divided by the interval duration—i.e., ${\langle {\left(\hat{x}\left(t\right)-{\langle \hat{x}\left(t^{\prime }\right)\rangle }_{t^{\prime }}\right)}^{2}\rangle }_{t}={\text{lim}}_{T\to \infty }\frac{1}{T}\int_{0}^{T}{\left(\hat{x}\left(t\right)-{\langle \hat{x}\left(t^{\prime }\right)\rangle }_{t^{\prime }}\right)}^{2}dt$; thus, we turn our attention to computing this integral. Importantly, because of its long time interval, the integral includes many sequences of the population reaching threshold, a variable amount of time until a first spike occurs, the production of a random number of spurious spikes during the spike propagation delay, and the return of the population to threshold; thus it effectively sums over all possible values of first-spike time and number of spurious spikes, with the values’ frequencies weighted by the probability distributions that we quantified above. To help simplify the integral, we approximate the timing of the spurious spikes by treating them as if they occur at the same time as the first spike because we are considering small delays *δ* ≪ *τ*; this is illustrated in Fig 2a by the lack of time differences between the random number of spikes, which are depicted together as a single vertical deviation at a single instant. Then performing the integration (see Section A.2.2 Mean readout error in S1 Appendix) reveals that we can express the combined effect of fluctuations from spike-time variability and spurious spikes via the sum of their variances—intuitively, one may expect these independent sources of variation to add in this way, as variances add for independent random variables in general; the integration yields the readout error *σ*_*readout*_ to leading order in small delay *δ* and small mean number of spurious spikes λ:

$$\begin{array}{cc} \sigma_{readout} & =\frac{1}{N}\sqrt{\frac{1}{12}+\frac{\delta^{2}}{\lambda^{2}\tau^{2}}+\lambda ,} \end{array}$$ (10)

where under the square root, the first term ($\frac{1}{12}$) arises from the baseline readout error in the case of zero delays and zero noise, the second term is the contribution from spike-time variability ($\frac{1}{\rho^{2}\tau^{2}}=\frac{\delta^{2}}{\lambda^{2}\tau^{2}}$ by the relation λ = *δρ*), and the third term is the contribution from the mean number of spurious spikes (λ). Importantly, we note that the mean number of spurious spikes λ can simply be thought of as a reparameterization of the probability rate *ρ*.

Furthermore, we can minimize Eq 10 with respect to λ to find the minimal readout error $\sigma_{readout}^{*}$ and optimal noise level, parameterized via the optimal mean number of spurious spikes λ*. This yields

$$\begin{array}{c} \lambda^{*}=2^{1/3}{\left(\delta /\tau \right)}^{2/3}\text{,} \end{array}$$ (11)

and

$$\begin{array}{c} \sigma_{readout}^{*}=\frac{1}{N}\sqrt{\frac{1}{12}+\frac{3{\left(\delta /\tau \right)}^{2/3}}{2^{2/3}}} \end{array}$$ (12)

$$\begin{array}{c} \sigma_{readout}^{*}\approx \frac{1}{N}[\frac{1}{\sqrt{12}}+\frac{3^{3/2}}{2^{2/3}}{\left(\delta /\tau \right)}^{2/3}]. \end{array}$$ (13)

We corroborate our analytic results with simulations in Fig 2b.

### Analysis of noise, delays, and coding error in the LIF model

In this section, we study the readout error *σ*_*readout*_ as a function of small delays and noise for the LIF model. We provide concise derivations here, and detailed derivations in S1 Appendix. For ease of exposition, we first examine the LIF model with zero delay (Δ = 0) and small noise (*σ* > 0) to isolate the contribution of spike-time variability to the readout error *σ*_*readout*_, as no spurious spikes can occur when spike propagation is instantaneous. Then, we will introduce and study small delay (Δ > 0). We will make use of several approximations and inequalities in our analysis of the LIF model, which will result in our final analytic expression for *σ*_*readout*_ being an approximate upper-bound on the actual error.

#### Readout error in the LIF model with no delays

To understand how the readout error *σ*_*readout*_ depends on small noise in the case of zero delay, we begin by describing the behavior of the population of membrane potentials under the dynamics of Eq 4 with delay Δ = 0. To gain some intuition, let us first consider what the membrane potential dynamics look like if spiking is disabled, i.e., the firing threshold *T* is taken to infinity. Importantly, the inhibitory terms −*τJ*_*ii*_*o*_*i*_(*t*) and $-\tau \sum_{j\neq i}^{N}J_{ij}o_{j}\left(t\right)$ are zero, and thus Eq 4 becomes the well-known Ornstein–Uhlenbeck (OU) process [25]. The stochastic process *V*_*i*_(*t*) after some time *t* ≫ *τ* approaches a stationary Gaussian process distribution with a mean of *N*/λ_*V*_ and a temporal autocovariance given by

$$\begin{array}{c} cov(V_{i}\left(s\right),V_{i}\left(t\right))≔\langle (V_{i}\left(s\right)-E\left(V_{i}\left(s\right)\right))(V_{i}\left(t\right)-E\left(V_{i}\left(t\right)\right))\rangle \end{array}$$ (14)

$$\begin{array}{cc} & =\frac{\sigma^{2}}{2\lambda_{V}}(e^{-\frac{\lambda_{V}}{\tau }\left|t-s\right|}). \end{array}$$ (15)

Thus at any moment of time, the membrane potentials are distributed as a Gaussian packet of constant width

$$\begin{array}{c} \sigma_{OU}≔\frac{\sigma }{\sqrt{2\lambda_{V}}}. \end{array}$$ (16)

and over a mixing time scale of *τ*/λ_*V*_, the membrane potentials diffuse and forget their past values due to the external noise. Note that we are interested in analyzing continuously operating networks, thus these stationary statistics will be relevant in our analysis.

Next, let us consider reintroducing the effects of spiking, returning to the original dynamics of Eq 4 with delay Δ = 0. We consider any initial condition in which the membrane potentials are all subthreshold with *V*_*i*_(0) < *T*, ∀*i*. As the membrane potentials travel toward threshold, driven by strong input current *I*_*i*_(*t*) = *N*, the top membrane potential will reach threshold first and spike. Importantly, this spike instantly, simultaneously, and uniformly inhibits all membrane potentials. Thus only the mean membrane potential is decremented, but the relative positions of the membrane potentials are preserved—i.e., the entire distribution is shifted lower by a constant value. Thus, this observation reveals a simple overall behavior: the membrane potentials are traveling in a Gaussian packet of constant width, with the entire packet being periodically decremented each time the top neuron in the packet reaches threshold and fires a spike.

**Fig 3:**
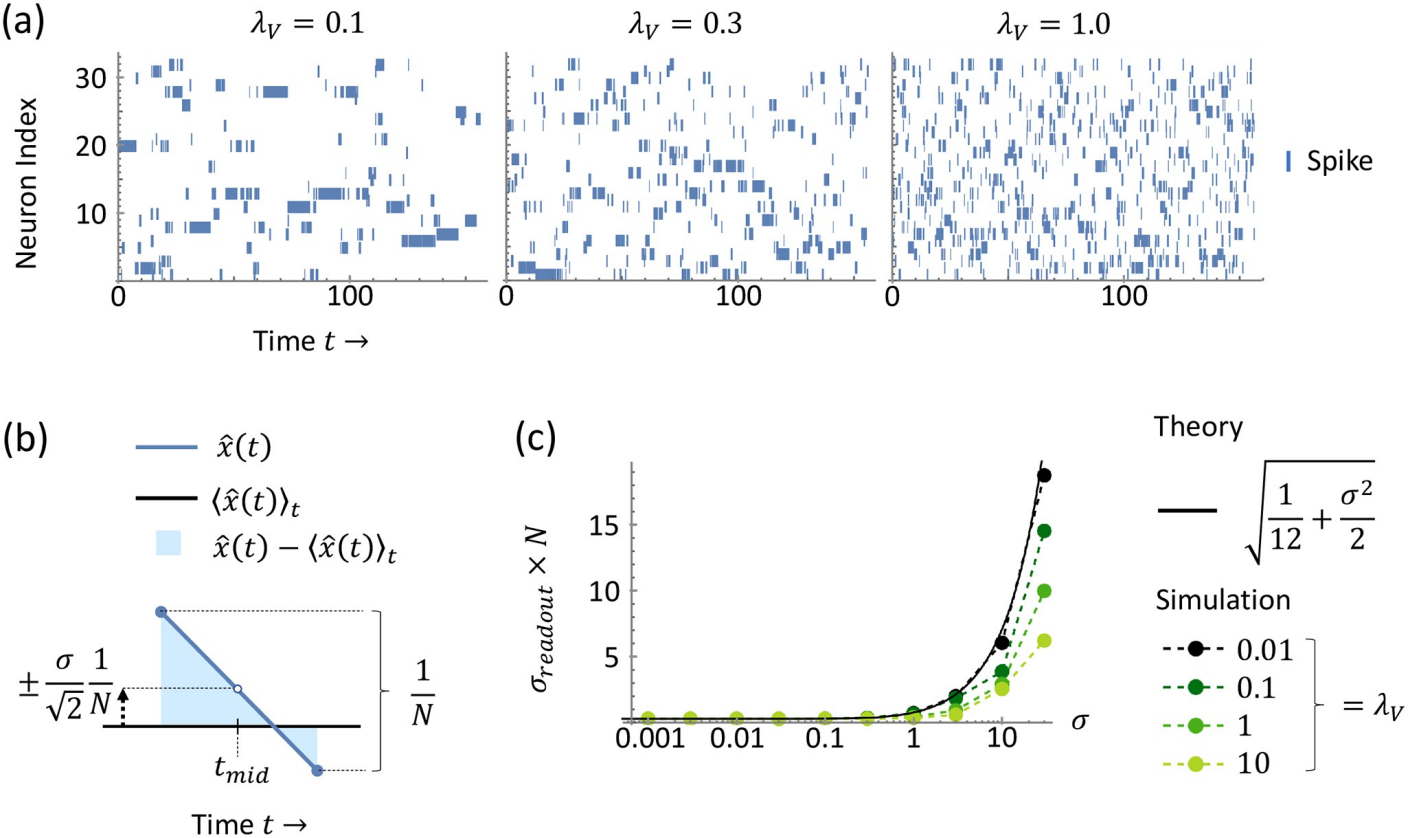
LIF model with zero delay. (a) Spike raster plots from simulations (*N* = 32) for three different values of the membrane potential leak λ_*V*_. Notably, for small λ_*V*_, we observe long runs in which the same neuron repeatedly spikes. Thus in the small λ_*V*_ limit, the next spike time is well-approximated by considering only the possibility that the same neuron spikes again. (b) Readout $\hat{x}\left(t\right)$ (blue) and its deviation $\hat{x}\left(t\right)-x\left(t\right)$ (light blue shaded) from the mean ${\langle \hat{x}\left(t\right)\rangle }_{t}$ for a single interspike interval. The variations in interspike interval durations accumulate to produce a variation in the readout with standard deviation $\frac{\sigma }{\sqrt{2}}\frac{1}{N}$, using the approximation from (a). (c) Readout error *σ*_*readout*_ as a function of noise *σ* for different values of λ_*V*_. Integrating the deviation illustrated in (b) across time yields the readout error in the small λ_*V*_ limit, Eq 19 (black). Simulations (dots on dashed lines, *N* = 64) with larger λ_*V*_ are upper-bounded by Eq 19.

With this membrane potential packet dynamics in mind, we will first quantify the spike-time variability of the first spike. It is important to understand this spike-timing variability as it contributes directly to the readout error *σ*_*readout*_ as we shall see below. We first start by considering the simplifying case of small λ_*V*_ ≪ 1. This limit affords two useful simplifications: (1) the membrane potentials fluctuate slowly within the Gaussian packet, with long mixing timescale *τ*/λ_*V*_ (Eq 15), and thus the same neuron repeatedly wins the race toward threshold (Fig 3a), and (2) the threshold-crossing time of this neuron is well-approximated by the first-passage time of ordinary Brownian motion with drift, as Brownian motion is simply an OU process with λ_*V*_ = 0. Thus on time-scales shorter than the long mixing time *τ*/λ_*V*_, we need only consider the dynamics of the neuron with the top membrane potential in the packet, as opposed to the entire population. Hence, we can express spike-time variability as random interspike interval durations, which are drawn from the Brownian motion first-passage time distribution. Importantly, the statistics of the first-passage time *t*_*fp*_ for Brownian motion are known [26]. For a particle undergoing Brownian motion with time constant *τ*, noise *σ*, drift *μ*, initial position *x*_0_, and the goal of reaching a threshold *θ*, the mean and variance of its first-passage time are $\langle t_{fp}\rangle =\frac{(\theta -x_{0})\tau }{\mu }$ and $\langle {\left(t_{fp}-\langle t_{fp}\rangle \right)}^{2}\rangle =\frac{\left(\theta -x_{0}\right)\sigma^{2}\tau^{2}}{\mu^{3}}$. In our case, the dynamics of the top membrane potential have time constant *τ*, noise *σ*, drift *I*_*i*_(*t*) = *N*, initial membrane potential of $-\frac{1}{2}$ (the threshold $T=\frac{1}{2}$ minus the self-reset of 1 through the −*τJ*_*ii*_*o*_*i*_(*t*) term in Eq 4), and the goal of reaching the threshold $T=\frac{1}{2}$. This yields the moments $\langle t_{fp}\rangle =\frac{\tau }{N}$ and $\langle {\left(t_{fp}-\langle t_{fp}\rangle \right)}^{2}\rangle =\frac{\sigma^{2}\tau^{2}}{N^{3}}$.

Now, to work toward calculating *σ*_*readout*_, which involves integrating $\hat{x}\left(t\right)$ over time, we start by considering the readout $\hat{x}\left(t\right)$ at a particular time *t*, under the influence of the spike-time variability we just quantified for small λ_*V*_. Recall that the readout $\hat{x}\left(t\right)$ is a uniform sum (*w*_*i*_ = 1) of instantaneous firing rates *r*_*i*_(*t*) (Eq 2) and that the firing rates *r*_*i*_(*t*) are simply leaky integrations of the spike-trains *o*_*i*_(*t*) (Eq 1). Thus we can write the readout $\hat{x}\left(t\right)$ as a sum of decaying exponentials, with one exponential for each spike-time *t*_*k*_ in the past:

$$\begin{array}{c} \hat{x}\left(t\right)=\frac{1}{N}\sum_{k=1}^{\infty }e^{-\frac{{\Delta t}_{k}}{\tau }}, \end{array}$$ (17)

where Δ*t*_*k*_ ≔ *t* − *t*_*k*_. Recalling that the interspike interval durations are random variables drawn from the first-passage time distribution, we recognize that the Δ*t*_*k*_ are also random variables, which are simply the sum over past interspike interval durations, $t_{fp}^{j}$:

$$\begin{array}{c} \Delta t_{k}=t-t_{1}+\sum_{j=1}^{k-1}t_{fp}^{j}. \end{array}$$ (18)

Now importantly, although the interspike interval durations $t_{fp}^{j}$ themselves are independent because the repeatedly-spiking top neuron always starts afresh at its reset potential after spiking and carries no history of the rest of the membrane potentials, we note that in contrast, the Δ*t*_*k*_ are correlated random variables because Δ*t*_*l*_ contains all the terms in Δ*t*_*k*_, ∀*l* > *k*. (Δ*t*_2_ contains all the terms in Δ*t*_1_, Δ*t*_3_ contains all the terms in Δ*t*_2_ and Δ*t*_1_, and so on.) Thus we have in $\hat{x}\left(t\right)$ (Eq 17) an infinite sum of correlated random variables. To evaluate the statistics of $\hat{x}\left(t\right)$, in particular its variance which contributes to *σ*_*readout*_, we make a simplifying approximation. Namely, by the central limit theorem, we take the sum $\sum_{j=1}^{k-1}t_{fp}^{j}$ in the Δ*t*_*k*_ (Eq 18) to be Gaussian because it contains many terms for most *k* in the sum from *k* = 1 to ∞ in $\hat{x}\left(t\right)$ (Eq 17). With this Gaussian approximation, the distribution of Δ*t*_*k*_ depends only on the mean and variance of *t*_*fp*_, as opposed to the more complex non-Gaussian first-passage time distribution.

Using this Gaussian approximation, we can calculate the variance of $\hat{x}\left(t\right)$ in Eq 17 in the limit of small noise, and we do so for a particular point in time *t*_*mid*_, halfway through a given interspike interval (illustrated in Fig 3b). We find that the variance in the readout $\hat{x}\left(t_{mid}\right)$ is $\frac{\sigma^{2}}{2N^{2}}$ (see Section A.3.1 Readout at a single point in time in S1 Appendix for a detailed calculation). Then finally to compute *σ*_*readout*_ (Eq 3), we integrate the squared deviation of the readout over a long time interval in the same manner as we did for the soft-threshold model (c.f. the paragraph preceding Eq 10). Namely, we recognize that this long interval of integration is simply comprised of many individual interspike intervals, and within each interspike interval, the deviations $\hat{x}\left(t\right)-{\langle \hat{x}\left(t\right)\rangle }_{t}$ are related to the variable $\hat{x}\left(t_{mid}\right)$, illustrated as a vertical shift of the readout $\hat{x}\left(t\right)$ in Fig 3b (note that *t*_*mid*_ here does not denote a single instant in time, but rather refers to the time in the middle of a given interspike interval). And since we have quantified the distribution of $\hat{x}\left(t_{mid}\right)$, we can perform the integration (see Section A.3.2 Mean readout error in S1 Appendix for details), which yields

$$\begin{array}{c} \sigma_{readout}=\frac{1}{N}\sqrt{\frac{1}{12}+\frac{\sigma^{2}}{2}}. \end{array}$$ (19)

We compare this expression to simulations, and we find empirically that it matches well for small λ_*V*_ (Fig 3c, λ_*V*_ = 0.01); this is to be expected because we used the simplifying case of small λ_*V*_ ≪ 1 in our derivation of Eq 19. However, importantly, releasing the assumption that λ_*V*_ is small, we find empirically that Eq 19 also serves as an upper-bound for general λ_*V*_ (Fig 3c, λ_*V*_ = 0.1, 1, 10).

#### Readout error in LIF model with delays

Next, we build upon our analysis for zero delay Δ = 0, and calculate the readout error *σ*_*readout*_ for small delay Δ > 0. The primary additional effect from the introduction of nonzero delay Δ is the possibility of spurious spikes. Spurious spikes increase the readout error *σ*_*readout*_, as we have seen in the soft-threshold model. Similarly, for the LIF model, we would like to quantify the statistics of spurious spikes and their contribution to the readout error *σ*_*readout*_.

**Fig 4:**
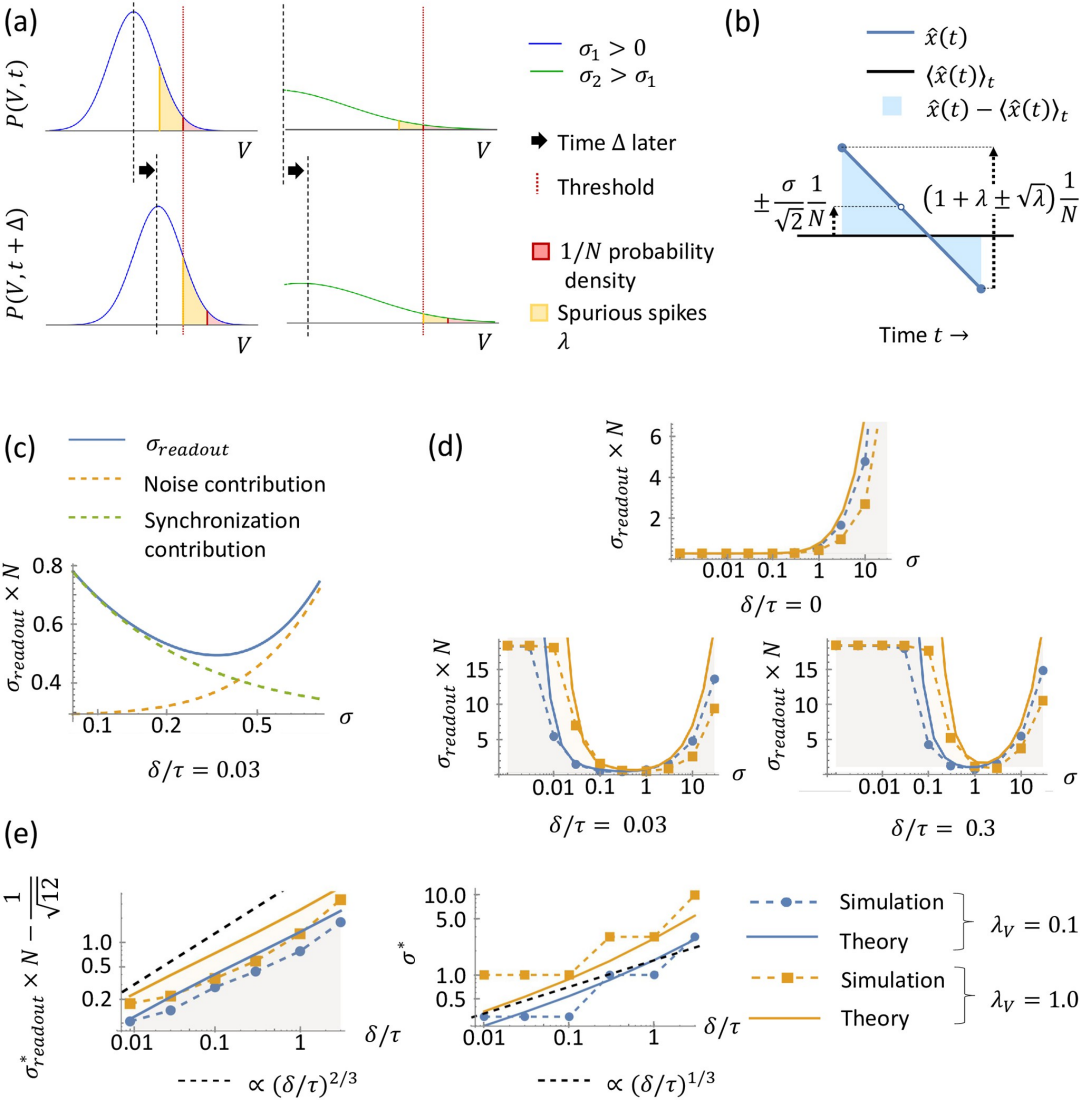
LIF model with nonzero delay. (a) Calculation of the number of spurious spikes. For a nonzero noise level *σ*_1_, the membrane potentials travel in a Gaussian packet with density *P*(*V*, *t*) (blue, top left) toward threshold (vertical red dotted line). The typical position of the packet at the time *t* when the first neuron spikes is determined by ensuring the tail probability (red, shaded area) above threshold equals 1/*N*. During the spike propagation delay Δ, the Gaussian packet continues traveling toward threshold (blue, bottom left), and the mean number of spurious spikes λ is given by the additional probability density that crosses threshold (yellow, shaded area). A larger noise level *σ*_2_ spreads out the Gaussian packet (green, right), thus reducing λ. (b) Readout $\hat{x}\left(t\right)$ (blue) and its deviation $\hat{x}\left(t\right)-x\left(t\right)$ (light blue shaded) from the mean ${\langle \hat{x}\left(t\right)\rangle }_{t}$. Similar to Fig 3b, the accumulated spike-time variation creates fluctuations in $\hat{x}\left(t\right)$ with standard deviation upper-bounded by $\frac{\sigma }{\sqrt{2}}\frac{1}{N}$, but in addition, spurious spikes introduce a Poisson variation in the readout with standard deviation $\sqrt{\lambda }\frac{1}{N}$. (c) Integrating the deviations illustrated in (b) yields an approximate upper-bound for *σ*_*readout*_, Eq 24 (blue). Conceptually, *σ*_*readout*_ receives contributions from noise (Eq 24 without the λ term; yellow, dashed), and synchronous spurious spikes (Eq 24 without the $\frac{\sigma^{2}}{2}$ term; green, dashed). (d) Readout error *σ*_*readout*_ for varying levels of noise. For zero delay (top), noise is not necessary to prevent spurious spikes, and thus it strictly increases *σ*_*readout*_. For non-zero delays (bottom), *σ*_*readout*_ has a U-shaped dependence on *σ*, and an optimal noise level σ* exists. The dots/squares on dashed lines represent *σ*_*readout*_ from simulations (*N* = 64), and solid lines are Eq 24, with the region below shaded, indicating upper-bound. Blue signifies λ_*V*_ = 0.1; yellow λ_*V*_ = 1.0. (e) Minimal readout error $\sigma_{readout}^{*}$ and optimal noise level σ* as a function of delay *δ*. Minimizing Eq 24 (with higher-order terms, see Eq S91 in S1 Appendix) with respect to *σ* yields $\sigma_{readout}^{*}$ (left, solid lines) and σ* (right, solid lines). $\sigma_{readout}^{*}-\frac{1}{\sqrt{12}}$ and σ* asymptotically approach (*δ*/*τ*)^2/3^ and (*δ*/*τ*)^1/3^, respectively (dashed black lines). We take the minimal *σ*_*readout*_ from the simulations in (d) and the associated optimal noise level to generate the dots/squares on the blue/yellow dashed lines, observing that our theory indeed provides an upper-bound for $\sigma_{readout}^{*}$ and a good estimate for the optimal noise level σ* in finite-sized simulations.

To calculate the statistics of spurious spikes, recall from our analysis for zero delay Δ = 0 that the membrane potentials travel in a Gaussian packet of width *σ*_*OU*_ (Eq 16) toward threshold, and eventually a top neuron in the packet reaches threshold and fires a spike. (See Section A.4.1 Mean number of spurious spikes in S1 Appendix for details on how small delays merely widen this packet slightly.) Then, during the spike propagation delay Δ, the other membrane potentials continue to travel toward threshold, and may fire extra, spurious spikes. We can estimate the mean number of spurious spikes during Δ by considering the tail of the approximately Gaussian membrane potential packet impinging upon threshold (Fig 4a).

The position of the Gaussian packet at the time of the first spike can be estimated via the condition that the tail probability above threshold *T* of the Gaussian packet is 1/*N*, so that out of *N* neurons the expected number of neurons to spike is 1 (Fig 4a, top). This tail probability condition approximately determines the location of the packet’s mean value $\bar{V}≔\frac{1}{N}\sum_{i=1}^{N}V_{i}$ via the condition

$$\begin{array}{c} \frac{1}{N}=\int_{T}^{\infty }\frac{1}{\sqrt{2\pi }\sigma_{OU}}e^{-\frac{{\left(V-\bar{V}\right)}^{2}}{2\sigma_{OU}^{2}}}dV. \end{array}$$ (20)

This condition can be solved to yield the approximate location of the mean $\bar{V}$ at the time of the first spike:

$$\begin{array}{c} \bar{V}=T-\sqrt{2}\sigma_{OU}\;{\text{erfc}}^{-1}(\frac{2}{N}), \end{array}$$ (21)

where erfc^−1^ is the inverse complementary error function.

Now we consider the mean number of extra spurious spikes that will occur in the time Δ after this first spike. Note that during this time, the Gaussian packet is moving up towards threshold at rate *N*/*τ*. Thus over a time Δ = *δ*/*N* all membrane potentials in the range *T* − *δ*/*τ* to *T* will further cross threshold (Fig 4a, bottom). Thus we can compute the mean number of spurious spikes λ by integrating the density of membrane potentials within this range:

$$\begin{array}{c} \lambda =N\int_{T-\delta /\tau }^{T}\frac{1}{\sqrt{2\pi }\sigma_{OU}}e^{-\frac{{\left(V-\bar{V}\right)}^{2}}{2\sigma_{OU}^{2}}}dV. \end{array}$$ (22)

Importantly, we recover the intuition that increased noise reduces the number of spurious spikes. Basically, increasing *σ* corresponds to larger *σ*_*OU*_, i.e. a wider packet, which in turn reduces the total density of membrane potentials in the range *T* − *δ*/*τ* to *T* that are ready to cross threshold after the first spike occurs. This can be readily understood by inspecting the leading-order expression for Eq 22 for small *δ*/*τ*, which is

$$\begin{array}{c} \lambda \approx c\left(N\right)\frac{\delta /\tau }{\sigma_{OU}}, \end{array}$$ (23)

where $c\left(N\right)≔\frac{Ne^{-{\left({\text{erfc}}^{-1}\left(2/N\right)\right)}^{2}}}{\sqrt{2\pi }}$ is a coefficient that grows sub-logarithmically with *N*, *c*(*N*) < *O*(log(*N*)). Thus interestingly, the total number of neurons *N* does not contribute strongly to the mean number of spurious spikes λ. Finally, since we are considering high-performing networks with a small mean number of spurious spikes (λ ≪ 1), we expect the number of spurious spikes to be well approximated by a Poisson distribution—the independent probability of each neuron crossing threshold during the delay gives rise to a binomial distribution for the number of spurious spikes, and a binomial distribution with a large number of trials and small per-trial probability (as is the case here) is well-approximated by a Poisson distribution. This completes our characterization of the spurious spike statistics.

Lastly, to calculate the readout error *σ*_*readout*_, we integrate the squared deviation in the readout $\hat{x}\left(t\right)$ across time, taking into account fluctuations due to: (1) spike-time variability from noise, which we isolated and quantified by analyzing the zero-delay case, and (2) the spurious spikes that we just characterized (Fig 4b). Evaluating the integral for *σ*_*readout*_ with the composition of these fluctuation sources (see Section A.4.2 Mean readout error in S1 Appendix for details), we obtain the approximate upper-bound

$$\begin{array}{c} \sigma_{readout}≲\frac{1}{N}\sqrt{\frac{1}{12}+\frac{\sigma^{2}}{2}+\lambda }, \end{array}$$ (24)

to leading order in *σ* and λ. Importantly, in this calculation we have used the fact that our result for *σ*_*readout*_ in the zero-delay case (Eq 19) was an approximate upper-bound (for general λ_*V*_, Fig 3c). Also we observed empirically that our calculation for the mean number of spurious spikes λ (Eqs 20 to 22) is an approximate upper-bound as well (see Section A.4.1 Mean number of spurious spikes in S1 Appendix). Thus the composition of these bounds on sources of fluctuation in turn provides an approximate upper-bound on readout error in Eq 24. A plot of our theory for the readout error as a function of noise *σ* for a fixed nonzero delay *δ* is shown in Fig 4c. We see that the readout error displays a non-monotonic dependence in the noise level *σ*, which arises as a trade-off between two competing effects. First, increasing noise contributes to increasing error through added spike-time variation through the middle term in Eq 24. But increasing noise *σ* also leads to a smaller mean number λ of spurious spikes through desynchronization of the population (see Eqs 23 and 16). This trade-off between reducing spike time variation and preventing spike synchronization leads to an optimal level of noise σ*.

We compare our approximate upper-bound for the readout error *σ*_*readout*_ against simulations, and we empirically observe that it indeed bounds the error and reproduces the expected dependence on *σ* and *δ* (Fig 4d). Further, we numerically minimize Eq 24 (with higher-order terms, see Eq S91 in S1 Appendix) to obtain an approximate upper-bound for the minimal error $\sigma_{readout}^{*}$ and an estimate for the associated optimal noise level σ* for a given delay *δ* (Fig 4e). Numerically differentiating our calculated $\sigma_{readout}^{*}$ and σ* with respect to *δ* (see Section A.4.2 Mean readout error in S1 Appendix), we find that they grow as

$$\begin{array}{c} \sigma_{readout}^{*}≲{\left(\delta /\tau \right)}^{2/3}, \end{array}$$ (25)

and

$$\begin{array}{c} \sigma^{*}∼{\left(\delta /\tau \right)}^{1/3}. \end{array}$$ (26)

Here, the growth of the minimal error $\sigma_{readout}^{*}$ (Eq 25) matches that of the soft-threshold model (Eq 12). And furthermore, the growth of the associated optimal noise level σ* also matches that of the soft-threshold model—the noise level in the soft-threshold model is the standard deviation of the time-to-spike, $\frac{1}{\rho }$, and Eq 11 implies that $\frac{1}{\rho *}∼{\left(\delta /\tau \right)}^{1/3}$.

## Discussion

In summary, we studied coding fidelity in tightly balanced networks of spiking LIF neurons with small noise and delays by analyzing the standard deviation *σ*_*readout*_ of a simple linear readout for a slowly-varying 1-D scalar dynamical variable. In contrast to previous works studying noise and delays in complex neural models chiefly via computer simulations, our work obtains a richer understanding by examining two simple noise modalities, the soft-threshold model and the LIF model, with simple finite transmission delays and deriving *analytical* expressions for the readout error *σ*_*readout*_ as a function of noise level and delay, revealing a power-law dependence on delay for the optimal noise level σ* and minimal readout error $\sigma_{readout}^{*}$.

For the soft-threshold model, we derived exact expressions (Eq 10) for *σ*_*readout*_ as a function of the delay and the superthreshold probabilistic spiking rate *ρ*, which we reparameterized as λ, the mean number of spurious spikes during a spike propagation delay—equivalent to an inverse noise level. For a given delay, we recovered the characteristic U-shaped dependence of *σ*_*readout*_ on λ (Fig 2). Minimizing our expression for *σ*_*readout*_ with respect to λ, we found that the optimal λ* grows with the delay as (*δ*/*τ*)^2/3^ and the associated minimal $\sigma_{readout}^{*}$ grows with the delay as (*δ*/*τ*)^2/3^ (Eqs 12 and 11).

For the LIF model, we characterized the dynamics of the membrane potentials as a Gaussian packet from an OU process impinging upon the threshold, and we derived an approximate upper-bound for *σ*_*readout*_ as a function of small noise and delays. Again, we recovered the characteristic U-shaped dependence of *σ*_*readout*_ on noise level (Fig 4). Minimizing our approximate upper-bound for *σ*_*readout*_, we found that $\sigma_{readout}^{*}$ grows with delay as (*δ*/*τ*)^2/3^ and the approximate optimal noise level σ* grows as (*δ*/*τ*)^1/3^ (Eqs 25 and 26). The behavior of $\sigma_{readout}^{*}$ matches that of the soft-threshold model, and the behavior of σ* matches the behavior of λ* in the soft-threshold model, when λ* is converted to a noise level.

Our hope is that our analytical results help quantitatively elucidate the fundamental mechanisms underpinning the beneficial role of noise in the presence of delays and provide a foundation for further analysis of more complex neural models. Indeed, our analytical results shed light on previous observations in simulations of more complex models, and they naturally suggest future directions of investigation. For example, in a neural model with more biophysical details than we consider in our work, [11] observes that the optimal noise level increases weakly with population size *N*, but the mechanisms underlying this phenomenon are not discussed. Interestingly, we can inspect our expression for the mean number of spurious spikes λ (Eq 23), and note that it has a coefficient *c*(*N*) that grows sub-logarithmically with population size *N*. Thus as population size *N* grows, with all else held constant, the mean number of spurious spikes λ increases weakly with *N*. To mitigate this increase, one expects a corresponding weak increase in the optimal noise level σ* (which then widens the membrane potential packet to compensate for the increased coefficient *c*(*N*) in the number of spurious spikes λ, Eq 23), just as [11] observes in simulations. Indeed, studying the parameter λ, the mean number of spurious spikes during a propagation delay, could yield fruitful insight in such computational studies.

### Generality and future directions

#### More complex dynamics

We derive our results in the context of encoding a slowly-varying input, but our results may also apply to emulating a slowly-varying dynamical system. Indeed, the predictive coding framework [7] provides a formulation to map an arbitrary linear dynamical system to the connectivity of a network of spiking LIF neurons. To understand how our results relate to this more general task, we note that in the predictive coding framework, efficient coding is facilitated by a set of fast, instantaneous synapses, whereas the underlying linear dynamical system is facilitated by a set of slow synapses with finite-timescale dynamics. Importantly, when small axonal transmission delays are introduced, the fast synapses undergo a major change—they go from instantaneous to non-instantaneous. And in contrast, the slow synapses undergo a relatively minor change—they are now slightly delayed, in addition to already being slow. Thus the main contribution of delay to modifying the dynamics of such networks [7], should arise primarily from the effect of the delay on the fast, not the slow synapses. It is precisely the effect of delay on fast synapses which we treat in our analysis here. Thus, our results could be used to describe the leading-order degradation due to delays in the more general scenario of emulating a linear dynamical system, with slowly-varying dynamics. We also note that the predictive coding framework [7] can be extended to emulate non-linear dynamical systems [27]; the non-linear dynamics are also facilitated by slow synaptic connections, and thus our approach may be extended to this setting as well, again by capturing the leading-order effect of delays on the fast synapses.

And while we consider encoding a positive, one-dimensional scalar signal, our results can also apply to encoding an arbitrary *D*-dimensional vector in $R^{D}$. Importantly, when coding for a *D*-dimensional signal in the predictive coding framework [7], each neuron codes for some direction in $R^{D}$. And when the coding error grows in any particular direction, the subpopulation of neurons tuned for that direction compete to spike and correct the error. Thus, our calculations may be adapted to approximately describe this scenario by replacing the parameter *N* → *N*_*eff*_, where *N*_*eff*_ is the size of the effective subpopulation actively participating in coding any particular direction.

However, for coding signed signals, we note that our analysis for positive-only coding directions (*w*_*i*_ = 1, ∀*i*) leaves out the possibility of a detrimental ping-pong effect. That is, if one of the neurons has a negative coding direction (if *w*_*i*_ = −1 for some *i*), a spike from a positively-coding neuron excites the negatively-coding neuron (and vice versa), which can initiate a volley of spurious synchronous spikes. Naturally, the ping-pong effect also arises when neurons have antipodal coding directions in $R^{D}$. But importantly, the ping-pong effect can be mitigated by introducing an auxiliary coding dimension to eliminate antipodal neurons [7], increasing the firing threshold, or by simply removing the problematic excitatory connections that support the ping-pong effect [28], and then our analysis still holds.

And finally for completeness, while in our analysis we considered encoding the particular *constant* input signal *x*(*t*) = 1, we can see how our analysis can also apply to slowly *time-varying* signals by considering encoding an input signal *x*(*t*) = *a*, where *a* is an arbitrary *O*(1) positive constant. Repeating the derivations presented in our analysis, but with the input *x*(*t*) = *a*, yields an approximate upper-bound on the readout error *σ*_*readout*_, analogous to Eq 24, for when a slowly-varying signal has value *a*. For the LIF model, the current *I*_*i*_(*t*) (Eq 6) obtains an additional factor of *a*, and consequently the lower limit in the integral for the mean number of spurious spikes λ (Eq 22) becomes $T-a\frac{\delta }{\tau }$. Intuitively, a small input current (small *a*) results in fewer membrane potentials reaching threshold during the spike propagation delay, and thus fewer spurious spikes occur; and vice-versa for a large input current (large *a*). As a corollary, the optimal noise level increases monotonically with *a*, as additional noise is only beneficial insofar as it counteracts increased pathological synchronization. Thus, for example, one could use our analysis to calculate a single noise level that performs well overall for an arbitrary slowly-varying input signal by averaging across the signal’s distribution, or one could introduce an adaptive mechanism that dynamically tunes the noise level to optimal, depending on the network’s estimate $\hat{x}\left(t\right)$. However, importantly, we note that if one chooses to simply fix the noise level to the optimal noise level associated with a particular input value, say e.g., *a* = *a*_*max*_, then our approximate upper bound for the readout error (Eq 24, with *x*(*t*) = *a*_*max*_ used in preceding derivations) still upper-bounds the readout error for when the input signal is less than *a*_*max*_—for values less than *a*_*max*_, there is simply more noise than is necessary to optimally desynchronize the network. Hence, the optimal noise level associated with *a*_*max*_ facilitates the aforementioned readout error bound for any slowly-varying input signal bounded by *a*_*max*_.

#### Other forms of heterogeneity

We studied the soft-threshold and membrane noise as specific mechanisms that can provide beneficial heterogeneity in spike-times which can prevent pathological synchronization leading to excess spurious spikes. However, other mechanisms may provide such a beneficial heterogeneity as well. As a first example, consider again coding for *D*-dimensional signals. In high dimension *D* ≫ 1, one can choose the neural coding directions in the predictive coding framework [7] such that few neurons are similarly-tuned. For example, if each neuron were allocated a random coding direction in $R^{D}$, the typical cosine angle between the tuning, or coding directions, of any pair of neurons would be $O(1/\sqrt{D})$, while number of neurons could become exponential in *D* before the *maximal* cosine angle between the coding directions of any pair of neurons exceeds a given *O*(1) threshold. With such a choice, the size *N*_*eff*_ of the effective subpopulation actively competing to correct the error in any particular direction becomes small, because few neurons are similarly tuned—there are fewer redundantly-coding neurons and thus a lesser propensity for spurious spikes, which improves coding fidelity. However, we note that details such as refractory period become important here, because the minimal subpopulation that codes nearest the direction of the error cannot spike continuously, thus other neurons with nearby coding directions must be recruited, expanding the active subpopulation. The beneficial effects of reduced redundancy for coding high-dimensional signals in the presence of delays has been observed in simulations [28], and extending our results to treat the details of particular choices of neural coding directions is an interesting future direction.

Many other sources of additional heterogeneity exist. For example, synaptic failures [1] provide a tunable source of noise—synapses are in general unreliable, but redundant synapses can be added to increase reliability, or different synapse morphologies can be used to achieve different levels of reliability. Indeed, synaptic failures have been observed to benefit coding in the presence of delays [11]. Modified spiking dynamics can also foster heterogeneity, such as the L2 penalty described in [7, 11, 12] where neurons self-reset themselves more strongly than they inhibit others, encouraging more diverse neural activity. Heterogeneous temporal filters have been shown to benefit efficient coding in spiking neural networks as well [29]. Imprecise connectivity, i.e., adding a frozen noise to the connectivity in Eq 5, can also provide a beneficial heterogeneity via chaotic fluctuations [30, 31] and has been observed to do so in the rate-version of the predictive coding framework [15]. Other biophysical details, such as refractory periods, distribution of transmission delays, distribution of synaptic dynamics, distribution of leak λ_*V*_, and per-neuron membrane noise levels, could all serve to provide spike-timing heterogeneity in a manner that prevents excess synchronization and spurious spikes. Our work provides a foundation upon which these mechanisms can be further studied in an analytic framework. More generally, our work reveals a conceptual framework whereby spike-timing heterogeneity, originating from either single neuron noise, imprecise connections, network level chaos, or other sources, can endow spiking neural networks with superior computational capabilities in the presence of transmissions delays, by preventing the build up of pathological synchrony.

## Supporting information

S1 Appendix Supporting derivations and simulation details. (PDF)
