# Supplementary material for: Optimal noise level for coding with tightly balanced networks of spiking neurons in the presence of transmission delays
Source: PLoS Comput Biol. 2022 Oct 17;18(10):e1010593. doi: 10.1371/journal.pcbi.1010593 (PMC9576105; doi:10.1371/journal.pcbi.1010593)
Supplement: S1 Appendix — (PDF) [file pcbi.1010593.s001.pdf]

S1 Appendix
Supporting derivations and simulation details

Contents

A Supporting derivations **1**
- A.1 Models . . . 1
- A.2 Readout error for the soft-threshold model . . . 4
  - A.2.1 Readout at a single point in time . . . 5
  - A.2.2 Mean readout error . . . 9
  - A.2.3 Additional variance from random interspersing of 2-spike events . . . 14
- A.3 Readout error for the LIF model with zero delay . . . 17
  - A.3.1 Readout at a single point in time . . . 20
  - A.3.2 Mean readout error . . . 24
- A.4 Readout error for the LIF model with delay . . . 28
  - A.4.1 Mean number of spurious spikes . . . 30
  - A.4.2 Mean readout error . . . 32

B Simulation details **36**
- B.1 Soft-threshold model . . . 37
- B.2 LIF model . . . 37
- B.3 Simulation code . . . 38

A Supporting derivations

A.1 Models

We consider a network of N leaky integrate-and-fire neurons whose membrane potentials undergo the dynamics

$$\tau\dot{V}_i(t) = -\lambda_V V_i(t) + N - \tau o_i(t) - \tau\sum_{j\neq i} o_j\left(t - \frac{\delta}{N}\right) + \sqrt{\tau}\sigma\eta_i(t), \text{ and} \tag{S1}$$

neuron i emits a spike when $V_i > \dfrac{1}{2}$.

V_i is the ith neuron's membrane potential, " $\dot{}$ " indicates a time derivative, λ_V controls the strength of the leak, N is the input current, $o_j(t)$ is the jth neuron's spike train (represented as a sum of Dirac δ functions), $\Delta = \frac{\delta}{N}$ is the spike propagation delay, σ controls the noise level, η_i are independent Wiener processes, and the firing threshold is $\frac{1}{2}$. Note that neurons self-reset instantaneously to $V_i = -\frac{1}{2}$. Note that the power of the time scale τ is chosen to ensure that each term in Eq. (S1) has the same units as membrane voltage, which here we take to be dimensionless. To see this note that a δ-function has units of inverse time (so multiplying any spike train $o_i(t)$, which is composed of δ-functions, by τ makes these terms dimensionless) and note that the Wiener process $\eta_i(t)$ obeys $\langle \eta_i(t)\eta_j(t')\rangle_t = \delta_{ij}\delta(t-t')$, implying that $\eta_i(t)$ itself has units of inverse square root of time. Thus we multiply the last term by $\sqrt{\tau}$ to ensure that the standard deviation σ has the same units as the dimensionless membrane voltage. Finally, note the leak term λ_V is also dimensionless.

Our goal is to derive an expression that describes the readout error, which we define as the standard deviation $\sigma_{readout}$ of the readout $\hat{x}(t)$, as a function of the number of neurons N, the delay δ, and the noise level σ.

$$\hat{x}(t) = \frac{1}{N}\sum_{i=1}^{N} r_i(t) \tag{S2}$$

$$\tau \dot{r}_i(t) = -r_i(t) + \tau o_i(t) \tag{S3}$$

$$\sigma_{readout} = \sqrt{\left\langle \left(\hat{x}(t) - \langle \hat{x}(t')\rangle_{t'}\right)^2 \right\rangle_t} \tag{S4}$$

We derive our central result—an approximate upper-bound for $\sigma_{readout}$ in the $\delta \ll \tau$ limit—in Subsection A.4. But before tackling the full complexity of this system, we study a simpler system that exhibits the same noise tradeoff in the presence of delay: a tight-balance network of soft-threshold neurons. For this soft-threshold dynamics, we derive an exact expression for $\sigma_{readout}$ in the large N, small delay $\delta \ll \tau$ limit. The neurons undergo the dynamics

$$\begin{aligned} &\tau \dot{V}_i(t) = N - \tau o_i(t) - \tau \sum_{j\neq i} o_j(t-\Delta), \text{ and} \\ &\text{neuron i emits spikes with probability rate } \rho(V_i) = \begin{cases} \rho, & V_i > \frac{1}{2} \\ 0, & V_i \leq \frac{1}{2} \end{cases}. \end{aligned} \tag{S5}$$

To understand the behavior of this model, consider a simple situation where the membrane voltages V_i of all the neurons are the same and equal to 0, as would be the case when there is no external input, and if there were an additional leak term to ensure all membrane potentials decay to 0. Then suppose the external input equal to N is turned on at time $t = 0$. All the membrane potentials will rise linearly at a rate N/τ and become superthreshold when they reach $V_i = \frac{1}{2}$ together at the same time $\tau/2N$. The entire population will remain superthreshold until the first neuron in the network spikes. Below, we will denote the time interval from the simultaneous superthreshold crossing

time to the time of first spike by the random variable t_{first}. At this time t_{first}, the membrane potential of the first neuron to spike will be immediately reset (i.e. decremented by 1). The rest of the network however, will first receive inhibition from this initial spike only at time $t_{\text{first}} + \Delta$, due to the transmission delay Δ. Of course during the entire time all membrane potentials are still increasing at a rate N/τ due to the external input. Thus, as long as the time $t_{\text{first}} + \Delta$ is less than τ/N, the entire population will be subthreshold again after responding to delayed inhibition from the first spike. More generally, we refer to the interval of time between the entire population crossing above threshold and then returning to subthreshold as a superthreshold interval. The smallest this superthreshold interval can be is the $t_{\text{first}} + \Delta$, assuming the delayed inhibition from a single spike is sufficient to return the entire population to subthreshold state. Of course it could be longer if delayed inhibition from multiple spikes is required to return the entire population to a subthreshold state. As we see below, we will be working with short delays Δ and high enough superthreshold firing rates ρ so that typically a single spike suffices.

To find interesting, high performing operating regimes of this model, we consider the statistics of spiking during a superthreshold interval. First, for any individual superthreshold neuron, the probability of emitting a spike in a short time interval of duration dt is $\rho \times dt$, and the time to the next spike of that neuron is exponentially distributed with mean $1/\rho$. Thus the mean inter-spike interval (ISI) of a single super-threshold neuron is $1/\rho$. However, if all N neurons cross threshold simultaneously, at some reference time $t = 0$, then the time t_{first} to the first spike in the entire network is exponentially distributed with mean $\frac{1}{N\rho}$. Now if any individual neuron spikes, it can potentially immediately return to a subthreshold state through its own reset mechanism that decrements the membrane voltage by 1. However we will be operating in a large N regime with a small number of neurons spiking between the first spike at t_{first} and the first network wide inhibition due to that spike at time $t_{\text{first}} + \Delta$. So for large N we can neglect the small number of neurons that may have returned to a subthreshold state due to their own spiking during this interval $[t_{\text{first}} \ldots t_{\text{first}} + \Delta]$, and simply assume all N neurons are superthreshold during this interval. Thus the superthreshold network ISI, defined to be the mean interval between any successive pair of spikes occurring anywhere in the network, is $\frac{1}{N\rho}$. So during any part of the superthreshold interval of duration equal to the delay Δ, the expected number of spikes to occur is a Poisson random variable with mean $\lambda = N\rho\Delta$. In order to ensure in the large N limit that a large number of spikes do not impact the readout before the first spike has a chance to inhibit the network, we therefore choose $\Delta = \delta/N$ where δ is $O(1)$. This ensures that $\lambda = \rho\delta$ is $O(1)$, and so $O(1)$ spikes occur between the time the first spike occurs at t_{first}, and the time that spike first has a chance to inhibit the network at time $t_{\text{first}} + \Delta$. Also, with this scaling of the delay Δ, the shortest possible superthreshold interval, on average is simply the sum of the mean of the time to first spike t_{first} (which is $\frac{1}{N\rho}$) plus the delay (which is $\Delta = \frac{\delta}{N}$). During this mean time, the membrane voltages, which are still integrating at a rate $\frac{N}{\tau}$, rise

above threshold by an amount $\frac{N}{\tau}(\frac{1}{N\rho}+\frac{\delta}{N}) = \frac{1}{\tau\rho}+\frac{\delta}{\tau}$. As long as this quantity is less than 1, then on average, delayed inhibition from a single spike is sufficient to end the superthreshold interval. This quantity will be less than 1 at small delays δ, and small mean time $\frac{1}{\rho}$ to first spike in a *single* neuron, all relative to the single neuron integration time τ.

In summary, if the population first becomes superthreshold at a reference time $t = 0$, then: (1) the mean of the time t_{first} to the first network spike is $\frac{1}{N\rho}$; (2) the mean time when the network first receives delayed inhibition from this first spike is $\frac{1}{N\rho} + \frac{\delta}{N}$; (3) the mean number of extra, or spurious, network spikes during this delay period is $\lambda = \rho\delta$; and (4) the final membrane voltage at the end of this period (right before the inhibition) is $\frac{1}{\tau\rho} + \frac{\delta}{\tau}$. We will focus our analysis on the following limits. First, in order to focus on regimes of good encoding performance with low error, we assume the mean excess number of spurious spikes $\lambda = \rho\delta \ll 1$. Second, for ease of analysis we focus on the regime in which once the network is superthreshold, a single spike can on average return the network to a subthreshold state, and moreover, the few neurons that do spike during the superthreshold interval and are reset, do not return to a superthrehsold state before the rest of the network becomes subthreshold. For the first condition, we require $\frac{1}{\tau\rho} + \frac{\delta}{\tau} = \frac{\delta}{\tau}(\frac{1}{\lambda}+1) \ll 1$ which implies $\frac{\delta}{\tau} \ll 1$ since we are already assuming $\lambda \ll 1$. Furthermore, consider a neuron that fired during the superthreshold state and was immediately reset to a subthreshold state corresponding to a membrane voltage that is below threshold by an $O(1)$ amount. Due to integration of the external stimulus at a rate $\frac{N}{\tau}$ it will return to a superthreshold state within a time $O(\frac{\tau}{N})$. As long as the delay $\Delta = \frac{\delta}{N}$ is much less than $\frac{\tau}{N}$, the entire population will become subthreshold before this reset neuron becomes superthreshold again. Thus, in the combined limit $\lambda \ll 1$ and $\delta \ll \tau$, the entire network operates in a simple fashion: all membrane potentials cross threshold together, a single first spike occurs, and then after a delay Δ the entire network becomes subthreshold again. Then after a time $O(\frac{\tau}{N})$ all the membrane potentials cross threshold simultaneously again. Interestingly as we see below, performance is actually best when the network behaves in this fashion.

A.2 Readout error for the soft-threshold model

First, we integrate Eq. (S3) to write the readout at a given time t as a sum of exponential kernels from all spikes in the past.

$$\hat{x}(t) = \frac{1}{N}\sum_{k=1}^{\infty} e^{-\frac{\Delta t_k}{\tau}}. \tag{S6}$$

Here, $\Delta t_k = t - t_k$ where t_k is the time of the k'th spike in the past from any neuron in the network. Note that the time between any successive pair of network spikes scales with N as $O(1/N)$. To see this, consider two possible cases. First consider the case where the entire population is superthreshold, and a spike occurs. After a delay of $\Delta = \delta/N$, this spike will inhibit the entire

network, causing the entire population to become subthreshold. However, before this happens, spikes can occur in any neuron at a rate $N\rho$ and therefore the typical ISI between any pair of spikes that occur in the same superthreshold interval is $O(\frac{1}{N\rho})$. Now consider the the second case: the final spike in one superthreshold interval and the first spike in the next superthreshold interval. The time interval between these two spikes decomposes into 3 parts: (1) the waiting time for network-wide inhibition to return to a subthreshold state; (2) the time it takes for the external stimulus to drive the population back to a superthreshold state; (3) the waiting time t_{first} for the first network spike to occur after becoming superthreshold. For (1), because we are working in a limit where a single spike can typically return the network to a subthreshold state, the waiting time for returning to a subthreshold state is at most the delay $\Delta = \frac{\delta}{N}$ from the last spike (and is shorter if there were earlier spikes in the same superthreshold event before the last spike). For (2), we note that the mean number of spikes in a superthreshold event is $1 + \lambda$ (the first spike plus the mean number λ of spurious spikes). Each of these spikes will eventually decrement the membrane voltage by an $O(1)$ amount. Because $\lambda \ll 1$, the membrane voltage of all neurons will never descend below threshold by more than an $O(1)$ amount, and because the membrane voltages integrate the external drive at a rate $\frac{N}{\tau}$, they will recover to a superthreshold state in a time $O(\frac{\tau}{N})$. Finally, for (3), the mean of the time t_{first} to the first network spike is $\frac{1}{N\rho}$. Thus the sum of these 3 intervals is $O(\frac{\delta}{N} + \frac{\tau}{N} + \frac{1}{N\rho})$, and is overall $O(\frac{1}{N})$.

A.2.1 Readout at a single point in time

In order to compute $\sigma_{readout}$, we need to compute the standard deviation in $\hat{x}(t)$ over a time window. To do so, we first compute the statistics of the readout at a particular point in time t_{thres}, defined as the time when the entire network reaches threshold together. We denote the readout at this time by $\xi := \hat{x}(t_{thres})$. To compute the mean and variance of ξ, we need to understand the statistics of the past times Δt_k of all previous spikes, as these times contribute to the readout through Eq. (S6). Figure A shows a schematic for the trajectory of the population of membrane potentials undergoing the dynamics Eq. (S5). Assuming the neural population starts with initial conditions $V_i = 0 \; \forall i$, the population reaches threshold together (the first threshold-crossing shown in the schematic). Let z_k denote the duration of time between this threshold crossing and the first spike anywhere in the network. As discussed above z_k is an exponentially-distributed random variable with mean $\frac{1}{N\rho}$. Thereafter, there is a delay time $\Delta = \frac{\delta}{N}$ during which the other $N-1 \approx N$ neurons remain superthreshold and continue to fire probabilistically. In a case where no extra spurious spikes occur, the rest of the membrane potentials rejoin the first-firing neuron's membrane potential after receiving the inhibitory spike. The population then continues toward threshold, and the process repeats. Occasionally (due to our assumption of small λ) one (or more) spurious spikes may occur. For example, in the third superthreshold interval in Figure A, two spikes occur at times z_2 and z_3 after the time the population crosses threshold. Each of these spikes will decrement the membrane

voltage of all neurons by 1, and the entire population will recover arriving at the next superthreshold crossing over a time proportional to the total number of spikes in the previous superthreshold interval.

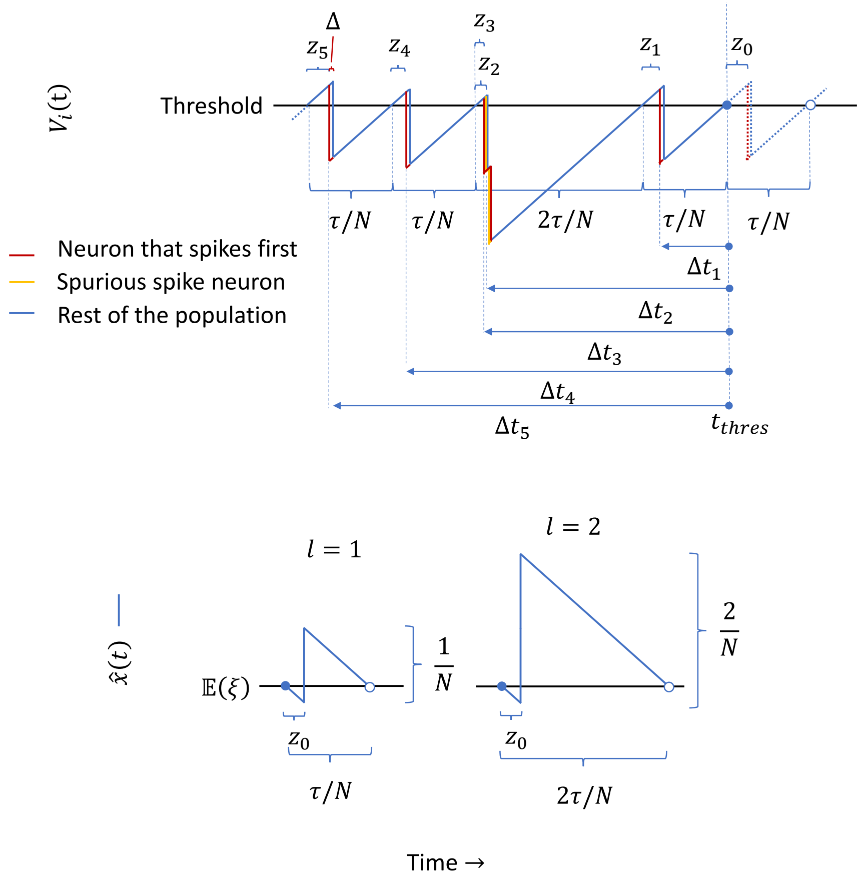

Figure A: Schematic for spike generation and readout error in the soft-threshold model. Top: the membrane potential dynamics Eq. (S5) generate a history of spike-times Δt_k relative to the moment the membrane potnetials reach threshold again, t_{thres}. The spike-times include variation z_k from the probabilistic first-spike wait-time due to the soft threshold, and the possibility of spurious synchronous spikes during the delay Δ. Bottom: integrating the readout deviation over l-spike events in the computation of $\sigma_{readout}$. $\mathbb{E}(\xi)$, the mean readout at the time t_{thresh}, is used as a reference point to compute the mean readout and the squared deviation from that mean, integrated over time.

For ease of exposition, let us first compute the mean and variance of ξ in the limit of extremely small λ. In this limit, the most probable scenario by far in each superthreshold interval, is that only a single first spike occurs, with no

subsequent extra spurious spikes. Later we will consider corrections due to extra spurious spikes that become relevant at larger λ. In this small λ limit, there is a one-to-one correspondence between individual spikes and the previous superthreshold crossing event of the population. We can use this correspondence to calculate the times $t_k = t_{thres} - \Delta t_k$ of the k spike in the past. First, let c_k be the time at which the population crossed threshold just before the time t_k of the k'th spike in the past. Thus by the definition of z_k we have $t_k = c_k + z_k$. Furthermore note that for any k the time between successive population superthreshold obeys $c_k - c_{k+1} = \frac{\tau}{N}$. To see this, note that by definition, at time c_{k+1} the membrane potentials are at threshold $V_i = \frac{1}{2}$. During the entire time from c_{k+1} to c_k, the membrane potentials are rising at a rate $\frac{N}{\tau}$, but they also suffer a decrement of 1 when the neuron that spiked resets its own membrane potential and when the rest of the neurons receive delayed inhibition from this spike. This combination of integration at a constant rate $\frac{N}{\tau}$, plus a decrement of 1, implies the next time the population crosses threshold will be at time $c_k = c_{k+1} + \frac{\tau}{N}$. This constancy of the time between population superthreshold crossings then implies that $c_k = t_{thres} - k\frac{\tau}{N}$ and so $t_k = c_k + z_k = t_{thres} - k\frac{\tau}{N} + z_k$, and thus $\Delta t_k = t_{thres} - t_k = k\frac{\tau}{N} - z_k$.

Inserting this formula for Δt_k into Eq. (S6), we obtain

$$\xi = \frac{1}{N}\sum_{k=1}^{\infty} e^{-\frac{k}{N}} e^{\frac{z_k}{\tau}}. \tag{S7}$$

We can see that ξ is an infinite sum of uncorrelated random variables. Using the fact that the variables $\frac{z_k}{\tau}$ are exponentially distributed with mean $\frac{1}{N\rho\tau}$, we have

$$\mathbb{E}(e^{\frac{z_k}{\tau}}) = \frac{N\rho\tau}{N\rho\tau - 1} \approx 1 + \frac{1}{N\rho\tau} \tag{S8}$$

$$var(e^{\frac{z_k}{\tau}}) = \frac{N\rho\tau}{(N\rho\tau-1)^2(N\rho\tau-2)} \approx \left(\frac{1}{N\rho\tau}\right)^2, \tag{S9}$$

where the approximate expressions denote the terms to leading orders in $\frac{1}{N}$. We can sum the geometric series, using the independence of the z_k, to obtain

$$\mathbb{E}(\xi) = \frac{1}{N}\sum_{k=1}^{\infty}(e^{-\frac{1}{N}})^k \frac{N\rho\tau}{N\rho\tau-1} \tag{S10}$$

$$= \frac{1}{N}\frac{N\rho\tau}{N\rho\tau-1}\frac{e^{-\frac{1}{N}}}{1-e^{-\frac{1}{N}}} \tag{S11}$$

$$\approx 1 + \frac{(2-\rho\tau)}{2\rho\tau}\frac{1}{N} + O\left(\frac{1}{N^2}\right) \tag{S12}$$

and

$$var(\xi) = \frac{1}{N^2}\sum_{k=1}^{\infty}(e^{-\frac{2}{N}})^k var(e^{\frac{z_k}{\tau}}) \tag{S13}$$

$$= \frac{1}{N^2} var(e^{\frac{z_k}{\tau}}) \frac{e^{-\frac{2}{N}}}{1-e^{-\frac{2}{N}}} \tag{S14}$$

$$= \frac{1}{2\rho^2\tau^2}\frac{1}{N^3} + O\left(\frac{1}{N^4}\right) \tag{S15}$$

We note some important properties of $\mathbb{E}(\xi)$ and $var(\xi)$. First, to leading order in $\frac{1}{N}$, $\mathbb{E}(\xi) = 1$, which is expected for the constant stimulus $x(t) = 1$. The next leading order term in $\mathbb{E}(\xi)$ decreases as $O(\frac{1}{N})$. Interestingly, in the limit of large ρ, the subleading term approaches $-\frac{1}{2N}$. This simple behavior can be understood as follows. In the large ρ limit, the network spikes very soon after it crosses superthreshold. This single spike increases the readout by exactly $1/N$. The readout subsequently decays at a rate τ but within a time $O(1/N)$ the membrane potentials recover to be subthreshold. By this time the readout has decayed to below 1 and is ready to be boosted to above 1 by the next spike. Thus, when we measure the readout specifically at the time the population has crossed threshold, before this next spike, the readout will tend to underestimate the input by an $O(1/N)$ amount, while right after the spike, the readout will overestimate the input by an $O(1/N)$ amount. In this fashion, the readout will zig-zag about the correct value. In fact it zig-zags symmetrically about the correct value (in the large ρ limit), and is thus $\frac{1}{2}\frac{1}{N}$ below 1 at threshold crossing and $\frac{1}{2}\frac{1}{N}$ above 1 just after the spike. In fact this zig-zag behavior of the readout cannot be reduced either by reducing delays (by reducing δ) or reducing variability in the time to first spike (by increasing ρ); it remains when $\delta = 0$ and ρ is large.

Further note that the standard deviation of the readout at the time the population crosses threshold (i.e. $\sqrt{var(\xi)}$ in Eq. (S15)) is $O(\frac{1}{N^{3/2}})$, and so is much smaller than the typical $O(1/N)$ amplitude of the zig-zag behavior of the readout about the correct value. Thus the variability in spike timing does not contribute appreciable variance to the readout ξ at the single time t_{thres}. This can be understood intuitively, as follows. First, the time to spike after a threshold crossing has variance $O(1/N^2)$ because N neurons are racing to spike first. However, even though the decoder only integrates over a time scale τ, the network generates spikes at a rate that is $O(N)$. Thus the decoder value ξ at time t_{thres} feels variability from the last $O(N)$ spikes in the past; the variabiilty in the timing of these spikes all contribute to the variance of the readout at t_{thres}. The combination of $O(N)$ spikes thus leads to an amplification of readout variance by a factor of N due to the addition law of variance of independent variables. But furthermore, the readout averages over N neurons leading to a multiplicative reduction of variance by $1/N^2$. Thus the total variance of the readout at any fixed time t_{thres} due to the combination of decoder averaging, decoder integration, and network racing to first spike is $O(\frac{1}{N^2}N\frac{1}{N^2}) = O(\frac{1}{N^3})$.

Note that we have shown that the standard deviation of ξ, or the readout at time t_{thres} is negligible and only $O(\frac{1}{N^{3/2}})$ in the small λ limit where only single spikes in any given superthreshold interval occur. Below, we will show that this conclusion still holds, even at larger λ when multiple spikes could occur with appreciable probability in a single superthreshold interval.

A.2.2 Mean readout error

So far, we have computed the mean and variance of the readout at a specific time t_{thres}. However, our fundamental goal is to compute the mean and variance of the readout across time. In doing so we will find that both the bias and the variance of the readout will be $O(1/N)$. To see this, consider integrating the readout across a long, contiguous time interval that starts at time $t = 0$ at the beginning of a superthreshold interval and that ends at the start of a much later superthreshold interval, at a time $t_{end} \gg \tau/N$. Then the mean readout is given by

$$\langle \hat{x}(t) \rangle_t := \frac{1}{t_{end}} \int_0^{t_{end}} \hat{x}(t) dt \tag{S16}$$

and with this definition, the bias of the readout is $\langle \hat{x}(t) \rangle_t - \langle x(t) \rangle_t = \langle \hat{x}(t) \rangle_t - 1$. The variance of the readout is given by

$$\sigma^2_{readout} := \frac{1}{t_{end}} \int_0^{t_{end}} (\hat{x}(t) - \langle \hat{x}(t) \rangle_t)^2 dt. \tag{S17}$$

Importantly, many superthreshold intervals occur in this long contiguous time interval from $t = 0$ to t_{end}. To see this, recall that each superthreshold interval contains $O(1)$ spikes because the mean number of spurious spikes $\lambda \ll 1$, and that a single spike inhibits the membrane potential population by 1. Thus after each superthreshold interval, it takes $O(1/N)$ time for the population to return to superthreshold because the membrane potentials are driven by an input current N/τ. For $t_{end} \gg \tau/N$ then, many superthreshold intervals occur in the time between $t = 0$ and t_{end}.

Now, to evaluate Eq. (S16) and Eq. (S17), we wish to express the trajectory of $\hat{x}(t)$ during and between these superthreshold intervals. Thus it is useful to introduce the notion of an "event", which we define to be the interval of time from the beginning of a superthreshold interval to the beginning of the subsequent superthreshold interval. By definition, each event contains a single superthreshold interval at its beginning. During this superthreshold interval, recall that a random number $l + 1$ spikes occur, where l is a Poisson random variable with mean λ. And after the superthreshold interval and before the end of the event, no further spikes occur since the population is subthreshold. Thus we can categorize events with the following nomenclature: events containing 1 spike are referred to as 1-spike events, events containing 2 spikes are referred to as 2-spike events, and so on. We can use this definition of event to simplify our primary task of integrating over the long time interval from $t = 0$ to t_{end} in Eq. (S16) and Eq. (S17) because the trajectory $\hat{x}(t)$ can be represented as

a sequence of events—one event starting after another, stitched together—and thus the integrals in Eq. (S16) and Eq. (S17) can be subdivided into a sum of many smaller integrals, with one integral for the duration of each event. Let M be the (large) number of events in the long time interval from $t = 0$ to t_{end}, then Eq. (S16) can be written as

$$\langle \hat{x}(t) \rangle_t = \frac{1}{t_{end}} \sum_{i=1}^{M} \mu_i \tag{S18}$$

where

$$\mu_i := \int_{t_i}^{t_{i+1}} \hat{x}(t) dt \tag{S19}$$

is the readout integrated from the i'th event's beginning, t_i, to its end, t_{i+1}. (The beginning of a subsequent event is the end of the preceding event, and the last event ends at time $t_{M+1} = t_{end}$.) Similarly, Eq. (S17) can be written as

$$\sigma^2_{readout} = \frac{1}{t_{end}} \sum_{i=1}^{M} s_i \tag{S20}$$

where

$$s_i := \int_{t_i}^{t_{i+1}} (\hat{x}(t) - \langle \hat{x}(t) \rangle_t)^2 dt. \tag{S21}$$

Now we must compute the sums of μ_i and s_i in Eq. (S18) and Eq. (S20), and to help us do so, we will take advantage of an important property of $\hat{x}(t)$ that we derived earlier. Namely, that the value of the readout at the time of threshold-crossing $\xi := \hat{x}(t_{thres})$ has $O(1/N^{3/2})$ fluctuations. Since we will soon see that the variance of the readout $\sigma_{readout}$ is $O(1/N)$, which is large compared to $O(1/N^{3/2})$, we can consider the value at the readout at threshold-crossing as effectively fixed at the value $\mathbb{E}(\xi)$. And since every event begins and ends at threshold-crossings, the readout at the beginning and end of each event is effectively fixed at the value $\mathbb{E}(\xi)$. Thus each μ_i and s_i do not depend on the history of $\hat{x}(t)$—the value of the readout at the endpoints of integration in Eq. (S19) and Eq. (S21) are always $\mathbb{E}(\xi)$ $(\hat{x}(t_i) = \mathbb{E}(\xi), \forall i)$, and the spike times in an event, relative to the start time of the event, are independent of all other events, as the superthreshold interval in the event produces spikes with spike-time distribution independent of other superthreshold intervals. Thus the μ_i are independent random variables and the s_i are independent random variables. (μ_i is independent of μ_j and s_j $\forall j \neq i$, and s_i is independent of μ_j and s_j $\forall j \neq i$; and of course, μ_i is correlated with s_i $\forall i$, because they are from the same event.)

With this independence, we are free to perform the sums in Eq. (S18) and Eq. (S20) in any order. We take advantage of this to group the events by their spike number, which we will see makes a further simplification. We write

$$\langle \hat{x}(t) \rangle_t = \frac{1}{t_{end}} \sum_{l=0}^{\infty} \sum_{i=1}^{Mp(l)+O(\sqrt{M})} \mu_i(l) \tag{S22}$$

$$\sigma^2_{readout} = \frac{1}{t_{end}} \sum_{l=0}^{\infty} \sum_{i=1}^{Mp(l)+O(\sqrt{M})} s_i(l) \tag{S23}$$

where

$$p(l) := \frac{\lambda^l e^- \lambda}{l!} \tag{S24}$$

is a Poisson distribution with mean λ—the probability for an event to have $l+1$ spikes. Here, we have written that there are $Mp(l)+O(\sqrt{M})$ $(l+1)$-spike events for each $l = 0, 1, ...$ because the number of $l+1$ spike events is a random variable with mean $Mp(l)$ and a deviation of order $O(\sqrt{M})$, as M is large, and we have defined the random variables $\mu_i(l)$ and $s_i(l)$ to have the same distribution as μ_i and s_i, but conditioned on the number of spikes in the event i being $l+1$. (The $O(\sqrt{M})$ deviation can be intuitively understood as the standard deviation from a binomial distribution, where a successful trial is an event having $l+1$ spikes, and a failure as having any other number of spikes.) Importantly, each $\mu_i(l)$ and $s_i(l)$ maintain independence from the other events because the conditional distribution is simply nomenclature. Since the $\mu_i(l)$ are independent random variables, we recognize the sum $\sum_{i=1}^{Mp(l)+O(\sqrt{M})} \mu_i(l)$ as an expectation value $\langle \mu_i(l) \rangle_i$ times $Mp(l) + O(\sqrt{M})$, where the angle brackets $\langle \cdot \rangle_i$ denote average over the distribution of $\mu_i(l)$. A parallel argument holds for $s_i(l)$, and we can write

$$\langle \hat{x}(t) \rangle_t = \frac{1}{t_{end}} \sum_{l=0}^{\infty} \left(Mp(l) + O(\sqrt{M}) \right) \langle \mu_i(l) \rangle_i \tag{S25}$$

$$\sigma^2_{readout} = \frac{1}{t_{end}} \sum_{l=0}^{\infty} \left(Mp(l) + O(\sqrt{M}) \right) \langle s_i(l) \rangle_i. \tag{S26}$$

which distills our task down to calculating $\langle \mu_i(l) \rangle_i$, $\langle s_i(l) \rangle_i$, and t_{end}.

Since we had replaced the integral over the long time interval from $t = 0$ to t_{end} in Eq. (S16) and Eq. (S17) with a sum over M events, we also need to express the normalizing fraction $\frac{1}{t_{end}}$ in terms of M so that we can eventually cancel out M that appears in Eq. (S25) and Eq. (S26)—M should not appear in the final result because we introduced it simply to express the long time window over which we are averaging. Fortunately, it is easy to express t_{end} by recalling that there are $Mp(l) + O(\sqrt{M})$ $(l+1)$-spike events $\forall l$ in the long time interval from $t = 0$ to t_{end}, and that each $l+1$ spike event has duration $(l+1)\tau/N$—each spike inhibits the membrane potential population by 1, yielding a total inhibition of $l+1$, and the input current N/τ constantly drives the membrane potentials back to threshold, counteracting the inhibition, ending the event when threshold is reached again at a time $(l+1)\tau/N$ after the start of the event. Thus we can simply sum up the duration of all events, to express $t_{end} = \sum_{l=0}^{\infty}(Mp(l)+O(\sqrt{M}))(l+1)\tau/N$, and we can substitute this expression into Eq. (S25) and Eq. (S26) to yield

$$\langle \hat{x}(t) \rangle_t = \frac{\sum_{l=0}^{\infty} \left(Mp(l) + O(\sqrt{M}) \right) \langle \mu_i(l) \rangle_i}{\sum_{l=0}^{\infty} (Mp(l) + O(\sqrt{M}))(l+1)\tau/N} \tag{S27}$$

$$\sigma^2_{readout} = \frac{\sum_{l=0}^{\infty}\left(Mp(l) + O(\sqrt{M})\right)\langle s_i(l)\rangle_i}{\sum_{l=0}^{\infty}(Mp(l) + O(\sqrt{M}))(l+1)\tau/N}. \tag{S28}$$

Now we can see that dividing both the numerator and denominator by M makes the M disappear from the expression: the terms with factor $Mp(l)$ become $Mp(l)/M = p(l)$, and the terms of order $O(\sqrt{M})$ become $O(1/\sqrt{M})$, which go to zero because M is large; thus dividing numerator and denominator by M yields

$$\langle \hat{x}(t)\rangle_t = \frac{\sum_{l=0}^{\infty} p(l)\langle \mu_i(l)\rangle_i}{\sum_{l=0}^{\infty} p(l)(l+1)\tau/N} \tag{S29}$$

$$\sigma^2_{readout} = \frac{\sum_{l=0}^{\infty} p(l)\langle s_i(l)\rangle_i}{\sum_{l=0}^{\infty} p(l)(l+1)\tau/N}. \tag{S30}$$

Finally, it remains to calculate the expectation values $\langle \mu_i(l)\rangle_i$ and $\langle s_i(l)\rangle_i$, which we recall are averages over the random variables Eq. (S19) and Eq. (S21), conditioned on there being $l+1$ spikes in the event in the time interval t_i to t_{i+1} in Eq. (S19) and Eq. (S21). Let us consider an instantiation of an $l+1$-spike event, compute $\mu_i(l)$ and $s_i(l)$ for this event, and then consider taking an average over instantiations of $l+1$-spike events to compute $\langle \mu_i(l)\rangle_i$ and $\langle s_i(l)\rangle_i$. Recall that for an instantiation of an $l+1$-spike event, the readout $\hat{x}(t)$ has value $\mathbb{E}(\xi)$ at the beginning and end of the event, that the time to first spike in the superthreshold interval at the beginning of the event is a exponentially-distributed random variable z_0 with mean $1/N\rho$ and standard deviation $1/N\rho$, and that l spikes occur during the delay Δ, after the first spike. We illustrate the readout's trajectory during this event in Figure A (bottom).

We highlight some important features of this trajectory of $\hat{x}(t)$ during the $l+1$ spike event. First, the readout $\hat{x}(t)$ is $\mathbb{E}(\xi) + O(1/N)$ during the entirety of the event, because each spike contributes $O(1/N)$ to the readout (Although we consider $l = 0, ..., \infty$ in Eq. (S27) and Eq. (S28), we recall that $\lambda \ll 1$, and thus $l > O(1)$ events are exponentially suppressed in $p(l)$, Eq. (S24).) And since the duration of the event is short, with duration $(l+1)\tau/N = O(1/N)$, compared to the decay of the readout, with time constant $\tau = O(1)$, the readout trajectory can be expressed as $(\mathbb{E}(\xi) + O(1/N))e^{-t'/\tau} = (\mathbb{E}(\xi) + O(1/N))(1 - t'/\tau + O(t'^2))$, where time t' is time since the start of the event (time t in $\hat{x}(t)$ is $t = t_i + t'$, where t_i is the start time of the event). Thus to first order in time, the readout decreases linearly during the event (with the exception of discontinuous increases due to spikes), with slope $-(\mathbb{E}(\xi) + O(1/N))/\tau$, which is $-1/\tau$ to leading order in N, because $\mathbb{E}(\xi)$ is 1 to leading order in N (Eq. (S12)). We do not consider higher-order corrections to this approximately linear decrease in the readout, because the constant slope $-1/\tau$ provides consistency with our assumption that the readout at the beginning and end of the event, $\hat{x}(t_i)$ and $\hat{x}(t_{i+1})$, are effectively fixed at the expectation value of ξ—the constant slope of $-1/\tau$ over the duration of the event $(l+1)\tau/N$ perfectly balances the contribution $(l+1)/N$ to the readout from the $(l+1)$ spikes, i.e. $0 = (l+1)/N - 1/\tau \times (l+1)\tau/N$, returning the readout to $\mathbb{E}(\xi)$ at the end of

the event—and the constant slope is sufficient to find $O(1/N)$ contributions to $\sigma_{readout}$ as we will see.

Second, the standard deviation of the first-spike time $t_i + z_0$ (where t_i is the start time of the event) is much larger than the variability of the l spurious spike-times that occur during the delay Δ; importantly, this fact will allow us to collapse the spike-times of the l spurious spikes to a single time for the purposes of calculating the most salient contributions to $\langle \hat{x}(t) \rangle_t$ and $\sigma_{readout}$, as a more finely-timed treatment would only provide higher-order corrections. To see why this fact is true, recall that we are working in the $\lambda = \delta\rho \ll 1$ limit. Dividing both sides of this inequality by $N\rho$, we arrive at $\delta/N \ll \frac{1}{N\rho}$, and substituting in $\Delta = \delta/N$, we have $\Delta \ll \frac{1}{N\rho}$. Recalling that the standard deviation of z_0 is $1/N\rho$, we see here that the standard deviation of z_0 is much larger than the delay Δ. Now, while the first spike time is exponentially distributed, for the spike-times of the l spurious spikes we must remember that the $l + 1$ spike event is an event that is conditioned on having l spurious spikes occur during the delay Δ. (This assumes, as we did earlier, that we are working in a regime in which the first spike's inhibition is sufficient to return the population to subthreshold after the delay Δ, preventing the possibility of further spikes until the population reaches threshold again, which marks the end of the event.) Thus these l spikes are not exponentially distributed like the first spike—indeed, they must occur between time $t_i + z_0$ and $t_i + z_0 + \Delta$. Regardless of the spike-time distribution in this interval, the l spurious spikes have spike-times that are bounded in a time interval of width Δ, which is much smaller than the standard deviation of z_0. Thus the chief contribution to spike-time variability for the l spurious spikes comes from z_0, and we can collapse the l spikes to the same spike-time $t_i + z_0$, which we illustrate in Figure A (bottom).

Using these features together—the constant slope of the readout decay and the collapsing of the l spurious spike-times—we can write down $\mu_i(l)$ and $s_i(l)$ for an instantiation of an $l + 1$ spike event:

$$\mu_i(l) = \int_0^{z_0} \left(\mathbb{E}(\xi) - \frac{t'}{\tau} \right) dt' + \int_{z_0}^{(l+1)\tau/N} \left(\mathbb{E}(\xi) + \frac{1}{N}(l+1) - \frac{t'}{\tau} \right) dt' \quad \text{(S31)}$$

$$s_i(l) = \int_0^{z_0} \left[\left(\mathbb{E}(\xi) - \frac{t'}{\tau} \right) - \langle \hat{x}(t) \rangle_t \right]^2 dt' + \int_{z_0}^{(l+1)\tau/N} \left[\left(\mathbb{E}(\xi) + \frac{1}{N}(l+1) - \frac{t'}{\tau} \right) - \langle \hat{x}(t) \rangle_t \right]^2 dt' \quad \text{(S32)}$$

where the first integral is from the beginning of the event to the time of the first spike, and the second integral is from the time of the first spike to the end of the event. We next want to average over instantiations i to obtain $\langle \mu_i(l) \rangle_i$ and $\langle s_i(l) \rangle_i$. Importantly, recall that z_0 is an exponentially distributed random variable with mean and standard deviation $1/N\rho$, so we must average over this distribution. Averaging over this distribution yields

$$\langle \mu_i(l) \rangle_i = (l+1)\frac{\tau}{N}\mathbb{E}(\xi) + \frac{1}{N^2}\frac{(l+1)(l\rho\tau + \rho\tau - 2)}{2\rho} \quad \text{(S33)}$$

$$\langle s_i(l)\rangle_i = \frac{1+l}{3N^3\rho^2\tau}$$
$$(6+\rho\tau(-3(1+l-2(\langle\hat{x}(t)\rangle_t - \mathbb{E}(\xi))n) + ((1+l)^2 -$$
$$3(1+l)(\langle\hat{x}(t)\rangle_t - \mathbb{E}(\xi))N + 3(\langle\hat{x}(t)\rangle_t - \mathbb{E}(\xi))^2N^2)\rho\tau))) \tag{S34}$$

Now we can calculate the mean readout $\langle\hat{x}(t)\rangle_t$ by substituting the expression for $\langle\mu_i(l)\rangle_i$ (Eq. (S33)) into the expression for $\langle\hat{x}(t)\rangle_t$ (Eq. (S29)), which yields

$$\langle\hat{x}(t)\rangle_t = \mathbb{E}(\xi) + \frac{1}{N}\frac{\lambda^2\rho\tau + 3\lambda\rho\tau + \rho\tau - 2\lambda - 2}{2(\lambda+1)\rho\tau}. \tag{S35}$$

And then we can calculate the variance of the readout $\sigma^2_{readout}$ by substituting the mean readout (Eq. (S35)) into the expression for $\langle s_i(l)\rangle_i$ (Eq. (S34)), and substituting Eq. (S34) into the expression for $\sigma^2_{readout}$ (Eq. (S30)), which yields

$$\sigma^2_{readout} = \frac{1}{N^2}\left[\frac{(\delta/\tau)^2}{\lambda^2} + \frac{1+\lambda(14+\lambda(19+\lambda(10+\lambda)))}{12(1+\lambda)^2}\right] \tag{S36}$$
$$= \frac{1}{N^2}\left[\frac{1}{12} + \frac{(\delta/\tau)^2}{\lambda^2} + \lambda + O(\lambda^2)\right], \tag{S37}$$

where we have used $\lambda = \rho\delta$ to substitute ρ for $\frac{\lambda}{\delta}$.

We can find the optimal noise level and minimal $\sigma_{readout}$ by differentiating $\sigma_{readout}$ with respect to λ and setting the derivative equal to zero. This yields

$$\lambda^* = 2^{1/3}(\delta/\tau)^{2/3} \tag{S38}$$
$$\sigma^*_{readout} = \alpha\sqrt{\frac{1}{12} + \frac{3(\delta/\tau)^{2/3}}{2^{2/3}}} \tag{S39}$$

We corroborate these results with simulations (Figure 2).

A.2.3 Additional variance from random interspersing of 2-spike events

Importantly, we had assumed in our calculations for the mean readout $\langle\hat{x}(t)\rangle_t$ and the readout fluctuations $\sigma_{readout}$ that the variance of the readout at the time of threshold-crossings $\xi := \hat{x}(t_{thresh})$ is to leading order $var(\xi) = O(1/N^3)$; we used this fact to consider the value of the readout at the start and end of events as effectively fixed, compared to the leading order fluctuations in $\sigma_{readout}$, which were $O(1/N)$. However, we had only shown $var(\xi) = O(1/N^3)$ if one considers that only 1-spike events occur, and no $l+1$ spike events occur (where $l > 0$) (the derivation leading to Eq. (S15)). So it remains to confirm that, when there is an appreciable probability that $l+1$ spike events occur (where $l > 0$), the variance $var(\xi)$ is still $O(1/N^3)$.

To see why this is the case, we first imagine a readout trajectory $\hat{x}(t)$ that consists only of 1-spike events, of which we know $var(\xi) = O(1/N^3)$; then

we modify $\hat{x}(t)$ so that it includes l-spike events, calling this new trajectory $\tilde{\hat{x}}(t)$; and finally we calculate how much the variance $var(\tilde{\xi})$ of the readout at threshold-crossing $\tilde{\xi} := \tilde{\hat{x}}(t_{thres})$ changes under the perturbation $\hat{x}(t) \to \tilde{\hat{x}}(t)$, compared to the original variance $var(\xi)$. If the change $var(\tilde{\xi}) - var(\xi)$ is an $O(1/N^3)$ amount, then the $var(\tilde{\xi})$ is $O(1/N^3)$ as well, since we already know $var(\xi)$ is $O(1/N^3)$.

Recalling that the mean number of spurious spikes $\lambda \ll 1$, the most prevalent type of event is a 1-spike event (0 spurious spikes), and the second-most prevalent type of event is a 2-spike event (1 spurious spike). Thus we can consider the perturbation $\hat{x}(t) \to \tilde{\hat{x}}(t)$ to leading order in λ by only considering the addition of 2-spike events. Now let us consider what adding 2-spike events looks like. The readout trajectory $\hat{x}(t)$ is a sequence of 1-spike events, while the readout trajectory $\tilde{\hat{x}}(t)$ is a sequence of 1-spike events and 2-spike events, with 1-spike events accounting for the fraction $p(0) = 1 - \lambda + O(\lambda^2)$ of all events and 2-spike events accounting for the fraction $p(1) = \lambda + O(\lambda)^2$ of all events. Importantly, the few 2-spike events are randomly interspersed among the many 1-spike events, as we recall that the number of spikes in a given event is independent of all other events. Thus, we can imagine adding 2-spike events to $\hat{x}(t)$ by randomly replacing a fraction λ of (randomly selected) 1-spike events with 2-spike events. Now importantly, 2-spike events have duration $2\tau/N$, while 1-spike events have duration τ/N, which means replacements must shift other events (events before the replacement to earlier times and/or events after the replacement to later times) in order to create room for the longer 2-spike event. Thus instead of considering this complicated dependency of the other event times due to each single replacement, let us instead consider replacing subsequent pairs of 1-spike events with 2-spike events; a subsequent pair of 1-spike events has duration $2\tau/N$, which matches that of a 2-spike event, and thus the other event times do not change when such a pair replacement is made.

To understand how many such replacements are necessary to result in a fraction λ of 2-spike events in $\tilde{\hat{x}}(t)$, consider starting with a large number M of events in a long time interval of $\hat{x}(t)$ as we did earlier. We wish to perform replacements of 1-spike events in $\hat{x}(t)$ so that the fraction of 2-spike events after replacements $\tilde{\hat{x}}(t)$ is λ. The question is—how many 1-spike pair replacements should we make? Let us consider replacing K 1-spike event pairs. The number of 1-spike events is initially M (there are only 1-spike events in $\hat{x}(t)$). Replacing K 1-spike pairs removes $2K$ 1-spike events and introduces K 2-spike events. Thus the number of 1-spike events becomes $M - 2K$ and the number of 2-spike events becomes K. We wish for the fraction of 2-spike events $\frac{K}{M-2K}$ to be λ. Solving for K, we find $K = M\lambda + O(\lambda^2)$. This means that we must replace a fraction $K/M = \lambda + O(\lambda^2)$ pairs of 1-spike events to make the fraction of 2-spike events to be λ.

Now that we know the fraction of 1-spike event pairs to replace with 2-spike events to represent the perturbation $\hat{x}(t) \to \tilde{\hat{x}}(t)$, we need to consider what the effect such a perturbation has on $var(\tilde{\xi}) - var(\xi)$. First, let us consider the replacement of one pair of 1-spike events, whose end is at a time $t_{replace}$,

which is $n\tau/N$ before the time t_{thresh} ($t_{replace} = t_{thresh} - n\tau/N$). Recall that the replacement only changes the spike times of the 2 spikes in the pair of 1-spike events, and the spike times in all other events remain the same. The perturbation in the readout at t_{thresh}, $\tilde{\xi} - \xi$, is

$$d(n) := \frac{1}{N}((e^{-(n+1)/N} + e^{-n/N}) - 2e^{-(n+1)/N}), \tag{S40}$$

where we have neglected any differences due to first-spike times because they are much smaller than the change due to the replacement. (The first-spike times in the original 1-spike events each have standard deviation $1/N\rho$, which is much smaller than the $O(\tau/N)$ jump in spike-time due to replacing the pair of 1-spike events with a 2-spike event. As discussed earlier, we are considering the regime in which a single spike's inhibition is sufficient to return the entire population to subthreshold, which means $1/N\rho \ll \tau/N$, and thus all spikes happen near the beginning of an event—the second spike here, which is originally in the second 1-spike event, now takes place near the beginning of the 2-spike event, being pushed back $O(\tau/N)$.)

Importantly, individual replacements are probabilistic, happening with probability λ for each pair of 1-spike events. Thus, now that we know the difference $\tilde{\xi} - \xi$ due to a replacement, we can use it to express how a single *probabilistic replacement* effects $var(\tilde{\xi}) - var(\xi)$. Consider any pair of 1-spike events in $\hat{x}(t)$ (indexed by n in their end time $t_{replace} = t_{thresh} - n\tau/N$); the probability that this n'th pair is replaced by a 2-spike event is λ. Thus the change in mean readout at t_{thresh}, $\mathbb{E}(\tilde{\xi}) - \mathbb{E}(\xi)$, due to this single probabilistic replacement is

$$\langle d(n) \rangle := d(n)\lambda + 0(1 - \lambda). \tag{S41}$$

where the change $d(n)$ due to the potential replacement is weighted by its probability λ. Furthermore, the contribution to the variance in the readout at t_{thresh}, $var(\tilde{\xi}) - var(\xi)$, due to this single probabilistic replacement is

$$var(d(n)) := (d(n) - \langle d(n) \rangle)^2\lambda + (0 - \langle d(n) \rangle)^2(1 - \lambda). \tag{S42}$$

where we have used the fact that the probabilistic replacement is independent of other sources of variance.

Finally, we must sum over all probabilistic replacements to express the total effect of 2-spike events on $var(\tilde{\xi}) - var(\xi)$. We can sum the additional variance from each probabilistic replacement $var(d(n))$, where each pair of 1-spike events considered for replacement is indexed by n in their end time $t_{replace} = t_{thresh} - n\tau/N$:

$$var(\tilde{\xi}) - var(\xi) = \sum_{n=1}^{\infty} var(d(n)) \tag{S43}$$

$$= -\frac{1}{N^2}\frac{e^{-2\alpha}(e^{1/N} - 1)(\lambda - 1)\lambda}{1 + e^{1/N}} \tag{S44}$$

$$= -\frac{1}{2}((\lambda - 1)\lambda)\frac{1}{N^3} + O(\frac{1}{N^4}). \tag{S45}$$

where we have safely neglected the low-probability event that two pairs of 1-spike events that share a 1-spike event (subsequent pairs of 1-spike events, overlapping in time) are both replaced, which has probability $O(\lambda^2)$. We can see that the leading-order term in Eq. (S45) is $O(1/N^3)$, which completes the argument that $var(\tilde{\xi}) = O(1/N^3)$ because we had already shown that the variance considering 1-spike events only, $var(\xi)$, is $O(1/N^3)$.

A.3 Readout error for the LIF model with zero delay

Our ultimate goal is to study the standard deviation $\sigma_{readout}$ as a function of the delay Δ and noise σ in the membrane potential dynamics Eq. (S1); as mentioned in Models, our study will uncover an upper-bound for $\sigma_{readout}$ as a function of delay Δ and noise σ. But for ease of exposition, let us first study $\sigma_{readout}$ in the case of zero delay $\Delta = 0$, with noise $\sigma > 0$. In this case, the membrane potentials undergo the dynamics

$$\tau \dot{V}_i(t) = -\lambda_V V_i(t) + N - \tau \sum_{j=1}^{N} o_j(t) + \sqrt{\tau}\sigma\eta_i(t), \text{ and} \\ \text{neuron } i \text{ emits a spike when } V_i > \frac{1}{2}. \tag{S46}$$

Studying this delay $\Delta = 0$ case, we will uncover properties of the membrane potential dynamics Eq. (S46) that will serve as a baseline for studying the dynamics when there is a delay $\Delta > 0$ (the dynamics in Eq. (S1))—we study $\Delta > 0$ in the next section.

To understand the behavior of the population of membrane potentials V_i in Eq. (S46), consider for the purpose of pedagogy the situation in which the membrane potentials start with some initial condition $V_i(0)$, and inhibitory spike terms are disabled ($o_j(t)$ terms are removed, and the neurons never spike, even when they surpass threshold). The dynamics become

$$\tau \dot{V}_i(t) = -\lambda_V V_i(t) + N + \sqrt{\tau}\sigma\eta_i(t). \tag{S47}$$

These dynamics correspond to an OU process [1, 2]—each membrane potential $V_i(t)$ undergoes an independent OU process with time constant τ, leak constant λ_V, drift term N, and independent noise $\sqrt{\tau}\sigma\eta_i(t)$. In particular if the process starts at a well specified initial condition $V_i(0)$, then its mean $\mathbb{E}(V_i(t))$ evolves in time as

$$\mathbb{E}(V_i(t)) = V_i(0)e^{-\frac{\lambda_V}{\tau}t} + \frac{N}{\lambda_V}\left(1 - e^{-\frac{\lambda_V}{\tau}t}\right). \tag{S48}$$

Notably, the initial condition $V_i(0)$ is exponentially forgotten on a time scale of $\frac{\tau}{\lambda_V}$ and the external drive N forces the mean to saturate at the value N/λ_V, again over a time scale of $\frac{\tau}{\lambda_V}$. Importantly, however, the temporal autocovariance of the stochastic process $V_i(t)$, is independent of the drift term:

$$cov(V_i(s), V_i(t)) := \langle (V_i(s) - \mathbb{E}(V_i(s)))\,(V_i(t) - \mathbb{E}(V_i(t)))\rangle \tag{S49}$$

$$= \frac{\sigma^2}{2\lambda_V}\left(e^{-\frac{\lambda_V}{\tau}|t-s|} - e^{-\frac{\lambda_V}{\tau}(t+s)}\right). \tag{S50}$$

where s and t are two times and the angle brackets $\langle \cdot \rangle$ denote an average over the stochastic trajectory η_i. The second term $e^{-\frac{\lambda_V}{\tau}(t+s)}$ reflects the non-stationarity of the autocovariance due to the definite initial condition at time 0, and for large times $s, t \gg \tau$ this non-stationary term vanishes. Let us consider large times, as we are interested in the setting in which the network is continuously operating. And for a time $s = t$, the first term $e^{-\frac{\lambda_V}{\tau}|t-s|}$ is 1, showing us that for a particular large time $t \gg \tau$, the variance of $V_i(t)$ is

$$var(V_i(t)) := \langle (V_i(t) - \mathbb{E}(V_i(t)))^2 \rangle \tag{S51}$$

$$= \frac{\sigma^2}{2\lambda_V} =: \sigma_{OU}^2. \tag{S52}$$

Thus, for an instant in time t, each $V_i(t)$ is a random variable with mean $\mathbb{E}(V_i(t))$ and variance $var(V_i(t)) = \frac{\sigma^2}{2\lambda_V}$, which we have also named σ_{OU}^2 for a more brief notation in future calculations. Furthermore, it is known that $V_i(t)$ is Gaussian-distributed (a property of the OU process). Thus a simple picture emerges here—for any moment in time $t \gg \tau$, the membrane potentials are Gaussian-distributed with variance $\frac{\sigma^2}{2\lambda_V}$, forming what we call a "packet" that is centered at $\mathbb{E}(V_i(t)) = N/\lambda_V$. Of course, the individual membrane potentials fluctuate as time goes on, but the packet remains the same size, with variance $\sigma_{OU}^2 = \frac{\sigma^2}{2\lambda_V}$.

Next, let us consider reintroducing the inhibitory terms $o_j(t)$, and describe the dynamics Eq. (S46). Compared to our preceding description of the dynamics without inhibitory terms, we see one difference: the drift term N is now counteracted by the inhibitory spike term $-\tau \sum_{j=1}^{N} o_j(t)$. The question is, what effect does this have on the evolution of the packet we previously illustrated? To answer this question, let us highlight one important property of the autocovariance equation describing the packet, Eq. (S50). Namely, that this equation does not depend on the initial condition $V_i(0)$. Then imagine the following: the packet of membrane potentials evolves according to our previous description, until one neuron reaches threshold. This neuron fires a spike, and, importantly, this spike is instantaneously delivered to all other neurons through the $-\tau \sum_{j=1}^{N} o_j(t)$ term (as the delay $\Delta = 0$), which simultaneously decrements the entire membrane potential packet. Since the decrement is simultaneous, the relative positions of the membrane potentials in the packet are preserved over that instant in time (but of course, the mean membrane potential is decremented). Thus the development of the autocovariance of the packet is preserved (it continues to grow or saturate to a steady state as in Eq. (S50)) because we can consider the new position of the packet after the spike to simply be a new set of initial conditions, and the autocovariance does not depend on the initial conditions. Of course, the mean membrane potential oscillates in a zig-zag fashion, driving upward due to the drift term N and being decremented with each spike, but the packet eventually grows to the same steady-state variance of $\sigma_{OU}^2 = \frac{\sigma^2}{\lambda_V}$.

Thus to summarize the dynamics of Eq. (S46), we have a Gaussian packet of membrane potentials $V_i(t)$ rising toward threshold at a rate N/τ (driven

by the input current N) and discouraged from deviating from zero due to the leak term $-\lambda_V V_i(t)$. Since we are interested in an operating regime where the network dynamics are driven by tight balance (the input current N term), let us assume the leak term $-\lambda_V V_i(t)$ is small compared to the input current N, i.e. $|\lambda_V V_i(t)| \ll N$; thus the input current N surely drives the packet to threshold. When the top neuron in the packet hits threshold at a time we call t_{thresh}, it fires a spike that instantaneously decrements the entire population of membrane potentials by 1 (the $-\tau \sum_{j=1}^{N} o_j(t)$ term integrates to 1 because of the time constant τ multiplying $\dot{V}_i(t)$ in Eq. (S46)). Then, the membrane potential packet continues traveling toward threshold, and the process repeats. Note that in this repeated process, the same neuron does not necessarily fire successively—the membrane potentials continuously fluctuate within the packet, and thus a different neuron can become the top neuron in the membrane potential packet at any time.

Importantly, we wish to consider high-performing networks, and it is helpful to identify what regime of parameters facilitates high performance, i.e. small readout standard deviation $\sigma_{readout}$. First, let us consider the noise level σ. The width of the membrane potential packet σ_{OU} is controlled by σ through $\sigma_{OU} = \sigma^2/2\lambda_V$. Thus for small σ, the packet width is small, and for larger σ, the packet width is large. Now, to understand how the width of the packet affects the readout standard deviation $\sigma_{readout}$, let us consider first small σ, such that $\sigma_{OU} \ll 1$. In this case, the fluctuation in the membrane potential of the top neuron in the packet is small because the packet itself is tight. Thus the time between spikes Δt from when the top neuron, say, neuron i, spikes at a time $t_{thresh,1}$, and when the top neuron, neuron j (neuron j is not necessarily the same neuron as neuron i), subsequently spikes again at time $t_{thresh,2}$ ($\Delta t := t_{thresh,1} - t_{thresh,2}$) has very little variation. To see this, recall that the inhibition from the first spike at time $t_{thresh,1}$ inhibits the membrane potential packet by 1. The packet then travels toward threshold due to the input current N (recall that we can ignore the leak term $-\lambda_V V_i(t) \ll N$). Now, if the packet is infinitely tight, i.e. $\sigma_{OU} = 0$, then it takes a time $\Delta t = \tau/N$ for the population to reach threshold again at time $t_{thresh,2}$, with no fluctuations in the time Δt. (a point packet is travelling toward threshold with constant current N and repeatedly inhibited) This results in perfectly regular interspike intervals of τ/N, which correspond to an the ideal zig-zag readout as discussed in the large ρ limit of the soft-threshold model—the best performance achievable, given that the readout is defined as a sum of firing rates that are filtered spike-trains Eq. (S2). Next, consider a small $\sigma > 0$, in which $\sigma_{OU} > 0$. Now the fluctuations in the membrane potential of the top neuron can be significant because the membrane potentials fluctuate within a packet of finite width; thus variations are introduced in Δt. These variations are a departure from the perfectly regular spike-times of the $\sigma = 0$ case, thus they increase $\sigma_{readout}$. Thus we find here that high-performing networks correspond to low levels of noise σ.

Importantly, the magnitude of the fluctuations in the interspike interval time Δt is controlled by the standard deviation of the membrane potential

packet $\sigma_{OU} = \sigma/2\sqrt{\lambda_V}$. Thus another way to achieve consistent interspike interval times, aside from small σ, is to choose large λ_V because this will make σ_{OU} small as well. But we cannot adjust λ_V arbitrarily, as we recall that we would like to consider values of λ_V that correspond to the regime in which the network dynamics are dominated by tight balance, i.e., $|\lambda_V V_i(t)| \ll N$. Now, importantly, we are considering $\sigma_{OU} \ll 1$, and the membrane potentials $V_i(t)$ exhibit $O(1)$ fluctuations due to decrements from inhibitory spikes when the top neuron reaches threshold—thus the combined effects of the packet's width $\sigma_{OU} \ll 1$ and the inhibitory spikes create an overall membrane potential dynamics that exist in an $O(1)$ dynamic range. Hence, the leak term $\lambda_V V_i(t)$ indeed satisfies $|\lambda_V V_i(t)| \ll N$, so long as λ_V scales less than $O(N)$. Let us assume that λ_V is a given property of the network dynamics that satisfies this constraint.

Equipped with an understanding of the membrane potential dynamics Eq. (S46) and a qualitative understanding of how noise σ affects the dynamics, let us work toward our goal of quantitatively expressing the readout $\hat{x}(t)$'s standard deviation $\sigma_{readout}$ as a function of the noise σ for a network with some given leak parameter λ_V. We start by recalling that the readout $\hat{x}(t)$ is a sum of decaying exponentials, with each term corresponding to a spike-time in the network:

$$\hat{x}(t) = \frac{1}{N} \sum_{k=1}^{\infty} e^{-\frac{\Delta t_k}{\tau}}. \tag{S53}$$

Here, we have written $\Delta t_k := t - t_k$ where t_k is the time of the k'th spike in the past from any neuron in the network. For ease of exposition, it is useful to define the notion of an "event" here as the interval of time between two subsequent spikes. Thus the time interval from t_2 to t_1 is an event, the time interval from t_3 to t_2 is an event, and so on. (Note that this is the same definition of "event" as in our description of the soft-threshold model, because spike-times are equal to membrane potential threshold-crossing times in the LIF model; also note that we only have 1-spike events here, as there are no delays.)

A.3.1 Readout at a single point in time

Now, we eventually want to compute the integral of $\hat{x}(t)$ over time to determine $\sigma_{readout}$ (Eq. (S17)), but to begin let us consider the readout $\hat{x}(t)$ at just one point in time, $t_{mid} := t_1 + \frac{\tau}{2N}$, that is near the middle of the event that starts at time t_1. Let us call the value of the readout at this time $\xi := \hat{x}(t_{mid})$. To see why this time t_{mid} is near the middle of the event, consider the typical duration of an event—the membrane potential packet has been inhibited by 1 from the spike at the beginning of the event, and the driving current N takes a time τ/N to push the packet back to threshold, if we ignore fluctuations in the membrane potential packet. Thus, $t_{mid} = t_1 + \tau/2N$ is about halfway through the duration of the event.

Now importantly, the spike times t_k are random variables that depend on the fluctuations of the membrane potentials due to the noise term $\sqrt{\tau}\sigma\eta_i(t)$ in

Eq. (S46), and so ξ is also a random variable that depends on the the spike times Δt_k through Eq. (S53). Thus we need to study the spike times Δt_k. To simplify our analysis, let us first consider small λ_V, where $\lambda_V \ll 1$. To understand how small λ_V simplifies the dynamics of the membrane potentials, first consider Eq. (S50). Notice that λ_V sets the time-scale of autocorrelations in membrane potential trajectory $V_i(t)$ to τ/λ_V, as seen in the first term of Eq. (S50). Thus a small λ_V corresponds to a long mixing time τ/λ_V of the membrane potential packet. In essence on time scales much less than that of τ/λ_V, the relative order of membrane potentials will be preserved. Note also that the time it takes for the mean of the packet to travel to threshold right after experiencing inhibition is τ/N. Thus if the mixing time τ/λ_V is much greater than the time to rise to threshold τ/N, from event to event the identity of the top-neuron that first spikes will largely be preserved for successive events. We can see this effect in spike raster plots from simulations of the membrane potential dynamics Eq. (S46) (Figure 3(a)). Notably, as λ_V is decreased, the same neuron repeatedly spikes; i.e., the same neuron remains the top neuron in the packet over many events. Thus for small $\lambda_V \ll N$, we can approximate the spike-time generation dynamics by only considering the dynamics of a single neuron's membrane potential—that of the top neuron.

Considering just the single membrane potential of the top neuron evolving according to Eq. (S46), we can easily calculate the statistics of event durations, i.e., the time between the last spike of the top neuron and its next spike. This will in turn allow us to calculate the statistics of Δt_k in Eq. (S53). Let us consider an event that starts at time t_s and ends at time t_f, and let us call the membrane potential of the top neuron $V_\alpha(t)$ (α is the index of the top neuron). At the instant of t_s, the membrane potential $V_\alpha(t)$ has just reached threshold, the top neuron spikes, and the membrane potential $V_\alpha(t)$ is decremented by 1 by the inhibitory spike. Next, the membrane potential $V_\alpha(t)$ is driving back toward threshold by the input current N, and experiences two other effects during its journey: the leak $-\lambda_V V\alpha(t)$ and the noise $\sigma\eta_\alpha(t)$. Since λ_V is small and $V_\alpha(t)$ is $O(1)$, we can neglect the leak term. But the noise term $\sqrt{\tau}\sigma\eta_\alpha(t)$ is accumulated in the integration of Eq. (S46) while the membrane potential $V_\alpha(t)$ travels toward threshold, and the total time $t_{fp} := t_f - t_s$ it takes for the top neuron to reach threshold is a random variable. Fortunately, we can recognize t_{fp} as the first-passage time of a particle undergoing Brownian motion with drift N, noise level σ, time constant τ, and with a goal that is a distance 1 away. The moments of t_{fp} are known [2]. The mean is

$$\langle t_{fp} \rangle = \frac{\tau}{N} \tag{S54}$$

and the variance is

$$var(t_{fp}) := \left\langle (t_{fp} - \langle t_{fp} \rangle)^2 \right\rangle = \frac{\sigma^2 \tau^2}{N^3}. \tag{S55}$$

Furthermore, the duration of the next event is simply another instance of the random variable t_{fp}, as the history of previous events is not recorded in any

way in the state of the top neuron. Thus, defining the random variable t_{fp}^n as the time between spike time t_{n+1} and spike time t_n, i.e., $t_{fp}^n := t_n - t_{n+1}$, we can write the spike times Δt_k in Eq. (S53) a time $t = t_{mid}$ as

$$\Delta t_k = t_{mid} - t_1 + \sum_{n=1}^{k-1} t_{fp}^n \tag{S56}$$

$$= \tau/2N + \sum_{n=1}^{k-1} t_{fp}^n, \tag{S57}$$

where by definition $t_{mid} - t_1 = \tau/2N$.

Now that the Δt_k are expressed as sums of random variables t_{fp}^n, we can consider evaluating the readout at time t_{mid}, $\xi = \hat{x}(t_{mid})$. We rewrite Eq. (S53) as

$$\xi = \frac{1}{N} \sum_{k=1}^{\infty} e^{-\left(\frac{\tau}{2N} + \sum_{n=1}^{k-1} t_{fp}^n\right)/\tau}. \tag{S58}$$

Here we can recognize that ξ is a sum of correlated random variables, because the exponent of the k'th term is a sum of the first $k-1$ t_{fp}^ns; for instance, the $k = 2$ term contains t_{fp}^1, and all $k > 2$ terms also contain t_{fp}^1. Furthermore, the first-passage times t_{fp}^n are not Gaussian-distributed [2]. Thus evaluating this sum in non-trivial, in contrast to our analysis for the soft-threshold model. However, to make evaluating this sum more tractable, we note one important property: in the vast majority of $e^{-(\dots)}$ terms, the sum $\sum_{n=1}^{k-1} t_{fp}^n$ itself contains many terms. (For only the first few $e^{-(\dots)}$ terms ($k = 1, 2, 3, \dots$), does the sum $\sum_{n=1}^{k-1} t_{fp}^n$ have few terms.) Thus using the central limit theorem, we can approximate the sum $\sum_{n=1}^{k-1} t_{fp}^n$ as a Gaussian distribution. Furthermore, this Gaussian distribution only depends on the mean and variance of the t_{fp}^n. Of course, even with this simplification, we still need to take into account that the $e^{-(\dots)}$ terms are correlated.

To formalize our central limit theorem approximation, let us rewrite Eq. (S58) as

$$\xi = \frac{1}{N} \sum_{k=1}^{\infty} e^{-\left(\frac{\tau}{2N} + \sum_{n=1}^{k-1} \tau/N + \gamma_n\right)/\tau}, \tag{S59}$$

where

$$\gamma_n \sim \mathcal{N}(0, \mathrm{std} = \frac{\sigma\tau}{N^{3/2}}) \tag{S60}$$

is a zero-mean Gaussian-distributed random variable with variance equal to that of t_{fp}^n. (We pulled out the mean of t_{fp}^n to create the τ/N term in $Eq.$(S59)).

Now recall that we are ultimately interested in computing the variance of the readout $\sigma_{readout}^2$, and so as a starting point, let us compute the variance of the readout at the particular time t_{mid}, $\sigma_\xi^2 := \langle(\xi - \langle\xi\rangle)^2\rangle$. We begin calculating σ_ξ^2 by making some simplifications. First, we pull out the common factor for all terms $e^{-\tau/2N}$, and we substitute in the simple relation $\sum_{n=1}^{k-1} \tau/N = (k-1)\tau/N$

to obtain

$$\xi = \frac{1}{N} e^{-1/2N} \sum_{k=1}^{\infty} e^{-((k-1)\tau/N + \sum_{n=1}^{k-1} \gamma_n)/\tau}. \tag{S61}$$

Second, we note that the sum $h(k) := \sum_{n=1}^{k-1} \gamma_n$ has zero terms for $k = 1$, i.e., $h(1) = \sum_{n=1}^{0} \gamma_n = 0$, one term for $k = 2$, i.e., $h(2) = \sum_{n=1}^{1} \gamma_n = \gamma_1$, and so on. Thus there is a mismatch between the term-number k and the number of γ_n in the sum for term k. We would like to remove this mismatch to make the expression simpler, so we therefore pull out the first $k = 1$ term in Eq. (S61), and re-choose the index $k \to k - 1$ so that it now starts at $k = 1$ with the term that contains one γ_n (that is, γ_1). This results in an equivalent expression to Eq. (S61),

$$\xi = \frac{1}{N} e^{-1/2N} + \frac{1}{N} e^{-1/2N} \sum_{k=1}^{\infty} e^{-(k\tau/N + \sum_{n=1}^{k} \gamma_n)/\tau}. \tag{S62}$$

Now, we can see that the first term in Eq. (S62) is constant and not a random variable, so the variance σ_ξ^2 does not depend on it, thus we can neglect it in our calculation of σ_ξ^2. Thus our goal is to compute the variance due to the other terms in Eq. (S62). Importantly, the $e^{-1/2N}$ that we factored from all terms is simply a multiplicative factor, thus if we define

$$\zeta := \sum_{k=1}^{\infty} e^{-\left(k\tau/N + \sum_{n=1}^{k} \gamma_n\right)/\tau} \tag{S63}$$

then the variance of ξ is the variance of ζ times $(e^{-1/2N})^2$, i.e., $\sigma_\xi^2 = (\frac{1}{N} e^{-1/2N})^2 \sigma_\zeta^2$. Keeping this conversion from σ_ζ^2 to σ_ξ^2 in mind, we can turn our focus to computing σ_ζ^2.

Looking at Eq. (S63), we need to understand how we will compute the variance of a sum of correlated random variables. We note that we can break down the k'th term into a factor $e^{-k/N}$ multiplying the random variable $e^{-\sum_{n=1}^{k} \gamma_n/\tau}$. And we can see that the random variable $e^{-\sum_{n=1}^{k} \gamma_n \tau}$ is log-normal distributed, because the γ_n are Gaussian-distributed and so is their sum. Thus we see that we have a sum of weighted, correlated log-normal random variables. Importantly, the variance of the sum depends on the variance of the individual correlated random variables (and how they are correlated), thus it is useful to introduce here a definition for the variance σ_0^2 of the log-normal random variable $e^{-\gamma_n/\tau}$:

$$\sigma_0^2 := e^{\sigma^2/N^3} (e^{\sigma^2/N^3} - 1). \tag{S64}$$

Now, we can actually compute the variance of σ_ζ^2 by adding variances and splitting according to the contributions from each γ_n to account for correlations. To do this, we introduce a simple approximation that is accurate for small $\sigma \ll 1$; namely, that the variance of the random variable $e^{-\sum_{n=1}^{k} \gamma_n/\tau}$ is approximately

equal to the variance of the random variable $\sum_{n=1}^{k} e^{-\gamma_n/\tau}$ for all k. Thus, using this approximation as we consider the contribution to the variance from each term in ζ, we encounter a sum of now uncorrelated random variables, with coefficients determined by the term number k. Namely, the first few terms when considering the variance of ζ are $e^{-1/N}e^{-\gamma_1/\tau}$, $e^{-2/N}(e^{-\gamma_1/\tau}+e^{-\gamma_2/\tau})$, and so on. Thus we can sum the coefficients for each independent random variable (each γ_n), and add the coefficient sums in quadrature to yield the variance σ_ζ^2. We write

$$\sigma_\zeta^2 = \sigma_0^2 \left[\left(\sum_{k=1}^{\infty} r^k \right)^2 + \left(\sum_{k=2}^{\infty} r^k \right)^2 + ... \right] \tag{S65}$$

where we have introduced the definition

$$r := e^{-1/N} \tag{S66}$$

for brevity.

Next, to simplify Eq. (S65), we can recognize that the sums $\sum_{k=j}^{\infty} r^k$ are geometric series, whose totals are $\frac{r^j}{1-r}$ and write

$$\sigma_\zeta^2 = \sigma_0^2 \left[\left(\frac{r}{1-r} \right)^2 + \left(\frac{r^2}{1-r} \right)^2 + \left(\frac{r^3}{1-r} \right)^2 + ... \right]. \tag{S67}$$

And factoring out $\frac{r^2}{(1-r)^2}$, we see that the result Eq. (S67) is itself a geometric series, with constant ratio r^2, which we can substitute for its total $\frac{1}{1-r^2}$, writing

$$\sigma_\zeta^2 = \frac{\sigma_0^2 r^2}{(1-r)^2} \left[1 + r^2 + r^4 + ... \right] \tag{S68}$$

$$= \frac{\sigma_0^2 r^2}{(1-r)^2} \frac{1}{1-r^2}. \tag{S69}$$

Thus finally we can substitute back in the definitions for r and σ_0^2 into Eq. (S69), and recall the conversion $\sigma_\xi^2 = (\frac{1}{N} e^{-1/2N})^2 \sigma_\zeta^2$ to obtain

$$\sigma_\xi^2 = \left(\frac{1}{N} e^{\frac{1}{2N}} \right)^2 \frac{e^{-2/N}}{(1-e^{-1/N})^2} \frac{1}{1-e^{-2/N}} e^{\sigma^2/N^3} (e^{\sigma^2/N^3} - 1) \tag{S70}$$

$$\sigma_\xi^2 = \frac{\sigma^2}{2} \frac{1}{N^2} + O\left(\frac{1}{N^3} \right). \tag{S71}$$

A.3.2 Mean readout error

To summarize the result Eq. (S71), it tells us that the value of the readout at a time $\tau/2N$ into an event, $\hat{x}(t_{mid})$, is a random variable with standard deviation $\sigma/\sqrt{2}N$ to leading order. This is a useful fact, because it gives us a starting point for calculating $\sigma_{readout}$, as we now illustrate. Recall from our discussion of

the soft-threshold model, that we can express the trajectory of $\hat{x}(t)$ as a series of events (here events go from spike to subsequent spike, while in the soft-threshold model events went from superthreshold crossing to superthreshold crossing), and we can express the integrals over time that define the mean readout $\langle \hat{x}(t) \rangle$ and the variance of the readout $\sigma^2_{readout}$ as sums of of smaller integrals over the duration of individual events (Eq. (S18), Eq. (S19), Eq. (S20), and Eq. (S21)). But importantly, in contrast to the soft-threshold model, the value of the readout $\hat{x}(t)$ during each event is not independent of all other events; in fact, for the LIF model, our calculation for Eq. (S71) shows spike-time variability accumulated over many spikes (the $\sum_{n=1}^{k} \gamma_n$) creates fluctuations in ξ that are $O(1/N)$. Indeed, individual spike-time variability (the standard deviation of each γ_n) is only $\sigma\tau/N^{3/2} = O(1/N^{3/2})$, which is much smaller than the $O(1/N)$ spike-time variability in the soft-threshold model (the standard deviation of the first spike time) that was required to produce fluctuations $\sigma_{readout} = O(1/N)$ in the soft-threshold model where spike-time variability does not accumulate. Recognizing this important property that the readout $\hat{x}(t)$ is correlated from event to event, we can still however use our result for σ_ξ (Eq. (S71)) to compute $\sigma_{readout}$ because we need to take a sum over many events, and addition is commutative. The sum for $\sigma_{readout}$ (Eq. (S20)) can be executed in any order—thus all we need to know is the distribution of the integrated squared deviation s_i for an individual event.

Thus, we can perform a parallel computation for $\sigma_{readout}$ to that in the soft-threshold model (Equation Eq. (S18) to Eq. (S37)), except here we only have 1-spike events. Eq. (S29) becomes

$$\langle \hat{x}(t) \rangle_t = \frac{\langle \mu_i \rangle_i}{\tau/N}, \tag{S72}$$

and Eq. (S30) becomes

$$\sigma^2_{readout} = \frac{\langle s_i \rangle_i}{\tau/N}, \tag{S73}$$

where we recall that the denominator in these equations expresses the mean duration of an event, which for LIF model we recall is simply $\langle t_{fp} \rangle = \tau/N$. To express the expectation values $\langle \mu_i \rangle_i$ and $\langle s_i \rangle_i$, we can first consider a particular instance of an event, integrate over the duration of this event to compute μ_i and s_i, and then take the average over i to yield $\langle \mu_i \rangle_i$ and $\langle s_i \rangle_i$. For a particular event then, we can write

$$\mu_i = \int_0^{t^i_{fp}} \left(\xi_i + \frac{1}{2N} - \frac{1}{\tau} t' \right) dt' \tag{S74}$$

$$s_i = \int_0^{t^i_{fp}} \left[\left(\xi_i + \frac{1}{2N} - \frac{1}{\tau} t' \right) - \langle \hat{x}(t) \rangle_t \right]^2 dt' \tag{S75}$$

where ξ_i is an instance of the random variable ξ, and the time t' starts at the beginning of the event with $t' = 0$. The expression in () in Eq. (S74) and

Eq. (S75) is the readout $\hat{x}(t')$. Importantly, $\hat{x}(t')$ starts with value $\xi_i + 1/2N$ at $t' = 0$, and decays approximately linearly with slope $-1/\tau$ (by the same reasoning for the constant slope of $-1/\tau$ in the soft-threshold model). Thus at time $t' = \tau/2N$, the readout equals ξ_i, as the definition of ξ_i requires. Finally, the readout continues to decay until the event ends at time $t' = t^i_{fp}$.

To evaluate $\langle \mu_i \rangle_i$, let us represent the deviation of ξ_i from $\mathbb{E}(\xi)$ with the unit Gaussian random variable y_i multiplied by σ_ξ. Eq. (S74) becomes

$$\mu_i = \int_0^{t^i_{fp}} \left(\mathbb{E}(\xi) + \sigma_\xi y_i + \frac{1}{2N} - \frac{1}{\tau} t' \right) dt' \tag{S76}$$

and its expectation value (averaging over y_i and t^i_{fp}) is

$$\langle \mu_i \rangle_i = \mathbb{E}(\xi) + O(1/N^{3/2}) \tag{S77}$$

because y_i has zero mean, the integral from $t' = 0$ to the mean t^i_{fp} at $t' = \tau/N$ evaluates to exactly $\mathbb{E}(\xi)\tau/N$, and any additional contributions from the deviations of t^i_{fp} from its mean τ/N are order $O(1/N^{3/2})$ (Eq. (S55)). Thus the mean readout Eq. (S73) to leading order is

$$\langle \hat{x}(t) \rangle_t = \mathbb{E}(\xi). \tag{S78}$$

This result can be understood intuitively by comparing to the nominal operation of the predictive coding framework (the large ρ limit discussed in the soft-threshold model). Intuitively, for high-performing networks (low noise), the network produces perfectly regular spikes, and thus the decaying readout $\hat{x}(t) \approx 1$ by tracing a zig-zag around the encoded variable $x(t) = 1$. Since each spike contributes $1/N$ to the readout, the value of the readout immediately before each spike is $1 - 1/2N$, so that when the spike arrives, the readout is $1 + 1/2N$. This centers the zig-zag perfect around $\hat{x}(t) \approx 1$, and notably, since the time between spikes is short ($O(1/N)$), the readout decays linearly to 1 at the mid-point time between subsequent spikes. Hence, $\langle \hat{x}(t) \rangle_t = \mathbb{E}(\xi)$ for high-performing networks.

Next, to evaluate $\langle s_i \rangle_i$, let us again introduce the random variable y_i and substitute into s_i Eq. (S78) to obtain

$$s_i = \int_0^{t^i_{fp}} \left[\left(\mathbb{E}(\xi) + \sigma_\xi y_i + \frac{1}{2} - \frac{1}{\tau} t' \right) - \mathbb{E}(\xi) \right]^2 dt' \tag{S79}$$

$$= \int_0^{t^i_{fp}} \left[\sigma_\xi y_i + \frac{1}{2N} - \frac{1}{\tau} t' \right]^2 dt'. \tag{S80}$$

Performing this integral and taking the expectation value over i (averaging over y_i and t^i_{fp}), we obtain to leading order

$$\langle s_i \rangle_i = \frac{\sigma^2 \tau}{N^3} \left(\frac{1}{12} + \frac{\sigma^2}{2} \right) \tag{S81}$$

where we have again neglected any higher-order contributions from the deviations of t^i_{fp} from its mean, $\langle t_{fp} \rangle = \tau/N$. Substituting the leading-order term in this expression into Eq. (S73) and taking a square root, we finally obtain

$$\sigma_{readout} = \frac{1}{N}\sqrt{\frac{1}{12} + \frac{\sigma^2}{2}}. \tag{S82}$$

Now importantly, we recall that we made two important assumptions to arrive at this result. The first was that $\lambda_V \ll 1$, and the second was that the sum of spike-time variations $\sum_{n=1}^{k} \gamma_n$ could be well-approximated as Gaussian with the central limit theorem and that any deviations from this approximation are small because only a few sums $\sum_{n=1}^{k} \gamma_n$ have a small number of terms (small k, while in the calculation, k ranges from 1 to ∞). Thus for small λ_V, we evaluate our Gaussian approximation by comparing our result Eq. (S82) against Monte Carlo simulations that only contain the top neuron (Figure B). Secondly, to understand how our result relates to networks with larger λ_V—when the top neuron alone is not sufficient to describe the network's dynamics—we perform simulations with a variety of values of λ_V. Interestingly, we find empirically that Eq. (S82) provides an upper-bound for $\sigma_{readout}$ (Figure 3c). This upper-bound is an important result that will be used in the next section.

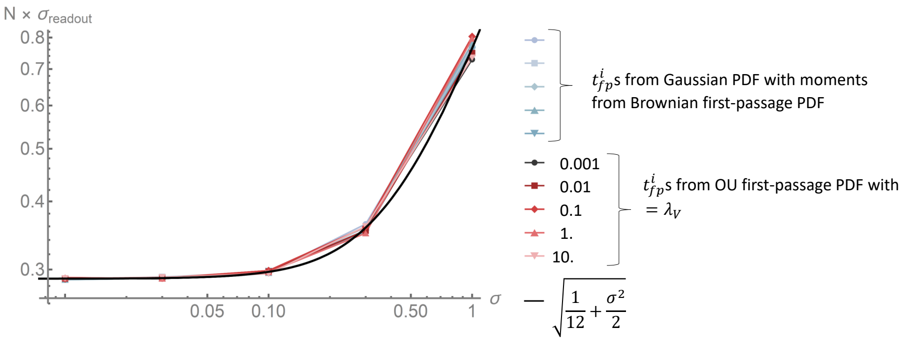

Figure B: The Gaussian approximation Eq. (S59) to the first-passage time distribution provides a good approximation for $\sigma_{readout}$ when compared to Monte Carlo simulations that contain only the top neuron. The standard deviation, $\sigma_{readout}$, of the readout (Eq. (S4)) when the t^i_{fp}s are drawn from the first-passage-time distribution of an OU process (red lines of different shades for different λ_V), appear to match $\sigma_{readout}$ when the t^i_{fp}s are drawn from a Gaussian distribution with moments from the first-passage-time distribution of Brownian motion (light blue lines, redundant simulations; black line, Eq. (S82)). These single-neuron simulations are equivalent to assuming the top neuron in the membrane potential packet remains the top neuron forever, like in the $\lambda_V \ll 1$ limit in Figure 3a.

A.4 Readout error for the LIF model with delay

In the previous section, we studied the dynamics Eq. (S1) with no delay (delay $\Delta = 0$) to determine the readout standard deviation $\sigma_{readout}$ as a function of the noise level σ. This provides a good starting point to determine $\sigma_{readout}$ as a function of both σ and nonzero delay $\Delta > 0$. To begin, let us consider the regime of parameters we would like to consider; in particular, we are interested in studying networks that exhibit high performance, i.e. small $\sigma_{readout}$, and networks that are driven by tight-balance, i.e., the leak term $-\lambda_V V_i(t)$ in Eq. (S1) is small compared to the driving current N. As discussed in the previous section, this regime corresponds to noise level $\sigma \ll 1$, and we consider λ_V as a given network property that satisfies the constraint that the leak term is small. Thus it remains to consider what values of delay Δ correspond to high performance. Intuitively, from our analysis of the soft-threshold model, we understand that small delays correspond to high performance—small delays allow one neuron to quickly inhibit the others, and this helps prevent spurious spikes that would otherwise increase readout error. Therefore, we consider small delays Δ for the LIF model. And more precisely, for ease of analysis, we will consider delays that are much smaller than the typical time between subsequent network spikes. The typical time between subsequent network spikes is $\langle t_{fp} \rangle = \tau/N$ (see previous section), and so we will consider delays $\Delta = \delta/N$, where $\delta \ll \tau$.

To understand how the membrane potentials evolve in the presence of delay $\Delta > 0$, let us first recall our description of the dynamics when $\Delta = 0$ from the previous section. The membrane potentials travel in a Gaussian packet of width $\sigma_{OU} = \sigma/\sqrt{2}\lambda_V$ toward threshold, driven by the input current N. When the top neuron in the packet hits threshold it fires a spike, and this spike then propagates to the other neurons, decrementing their membrane potentials, and thus prevents the other neurons from firing (until the packet reaches threshold again). Now this is where we see a departure from these dynamics due to the propagation delay Δ in Eq. (S1). The top neuron indeed fires a spike and resets itself when it reaches threshold (through the self-reset $-\tau o_i(t)$ term), but the spike takes a time Δ to decrement the other membrane potentials (through the $-\tau \sum_{j \neq i} o_j(t - \Delta)$ term). Thus the other neurons in the packet continue to travel toward threshold while they are waiting to receive the top neuron's inhibitory spike, and they may themselves cross threshold and fire spurious spikes. We will see, just as we saw in the soft-threshold model, that spurious spikes increase the readout error $\sigma_{readout}$, and thus one of our goals in this section will be to calculate the number of spurious spikes and the effect they have on the readout.

Now as we continue to describe the dynamics with delay $\Delta > 0$ and eventually calculate quantities like the mean number of spurious spikes, we will see as in the previous section, that defining the notion of an "event" is helpful. Thus we define an event here as the interval of time from when a top neuron in the membrane potential packet reaches threshold, at a time we refer to as $t_{thresh,1}$, to the next time a top neuron reaches threshold again (this latter top

neuron need not be the same as the previous top neuron), at a time we refer to as time $t_{thresh,2}$. Importantly, we define neurons that spike within the time interval $t_{thresh,1}$ to $t_{thresh,1} + \Delta$ as spuriously spiking neurons, and do not consider these neurons as candidates for marking the end of the event at $t_{thresh,2}$. Instead, the neuron that next spikes after $t_{thresh,1} + \Delta$ is denoted as the next top neuron to reach threshold, marking the end of the event when it reaches threshold at time $t_{thresh,2}$. To understand why this notion of event is well-defined, recall that we are focusing on the limit of small noise and small delays. In particular, note that the inhibition from one or more spikes (the first spike from the top neuron at $t_{thresh,1}$ and any additional spurious spikes that may occur) decrements the membrane potentials by 1 or more, and the membrane potentials are driven toward threshold by the current N. Thus it takes a mean time of $O(\tau/N)$ for the next spike to occur after $t_{thresh,1} + \Delta$ (the time when the entire population is first inhibited due to delayed inhibition from the first spike from the top neuron), with deviations from this mean time being small because we are considering the limit of small noise. And since we are considering delays $\delta \ll \tau$, which is equivalent to $\Delta \ll \tau/N$, we see that the time interval during which spurious spikes can occur is much smaller than the $O(\tau/N)$ time for the next nonspurious spike to occur. Thus there is a clear separation between the spike times of spurious spikes (between $t_{thresh,1}$ and $t_{thresh,1} + \Delta$) and the spike of the next top neuron $t_{thresh,2} = t_{thresh,1} + O(\tau/N)$.

Importantly, examining the interval from $t_{thresh,1}$ to $t_{thresh,1} + O(\Delta)$ more closely, we see another departure from the zero-delay dynamics described in the previous section when we consider that in any particular event, the top neuron is decremented at time $t_{thresh,1}$, while the other $N - 1$ neurons are decremented at time $t_{thresh,1} + \Delta$. (And if spurious spikes occur in the event, further differences in inhibition times in the population are of $O(\Delta)$.) Interestingly, this creates a "head start" toward threshold for the top neuron in the packet, relative to the other neurons, as the membrane potentials travel toward threshold until the next top neuron spikes at time $t_{thresh,2}$. To see this, recall that the top neuron's membrane potential is reset to $-1/2$ when it self-resets at time $t_{thresh,1}$. At this point in time, it is receiving an extra small, excitatory current from the leak term $-\lambda_V V_i$ because V_α (where α is the index of the top neuron) is negative—this is in addition to the input current N. Meanwhile, the other membrane potentials are still traveling toward threshold, and the membrane potentials near the top of the packet are near the threshold at $1/2$. Thus these neurons experience a small, additional inhibitory current from the leak term $-\lambda_V V_i(t)$, slightly slowing their travel toward threshold. So we see that after all membrane potentials have received the inhibitory spike after delay Δ, the top neuron has integrated extra current relative to the other neurons near the top of the packet, and so it has a head start on its way toward threshold. This corresponds to a stretching of the upper tail of the membrane potential packet from the nominal Gaussian distribution of width σ_{OU}. We will return to this subtle but important departure from a Gaussian packet when the nature of the packet's tail becomes important in our calculations.

A.4.1 Mean number of spurious spikes

Equipped with our understanding of the dynamics Eq. (S1) and the definition of an event, let us now turn to quantifying the number of spurious spikes in a given event, as spurious spikes increase the readout error and thus must be included in our study of $\sigma_{readout}$. With the objective of quantifying the number of spurious spikes in an event, consider an event that starts at time $t_{thresh,1}$ and ends at time $t_{thresh,2}$. At time $t_{thresh,1}$, the top neuron in the membrane potential packet has just reached threshold, and the rest of the neurons in the packet are below threshold. At time $t_{thresh,1}$, we can estimate the typical position of the mean of the membrane potential packet, which we denote as $\bar{V}$, by computing what the value of the mean must be so that the tail of the membrane potential distribution that is above threshold is $1/N$. When this tail probability is above $1/N$ it is likely that out of the N neurons, the top neuron has just crossed threshold. The probability density of the membrane potential packet is approximately Gaussian with width σ_{OU}. Thus the approximate position of the mean $\bar{V}$ of the membrane potential packet relative to the threshold, i.e. $\theta := 1/2 - \bar{V}$, is then estimated via the condition

$$\frac{1}{N} = \int_{\theta}^{\infty} \frac{1}{\sqrt{2\pi}\sigma_{OU}} e^{-\frac{z^2}{2\sigma_{OU}^2}} dz, \tag{S83}$$

or equivalently,

$$\theta = \sqrt{2}\sigma_{OU} \ \mathrm{erfc}^{-1}\left(\frac{2}{N}\right). \tag{S84}$$

Next, we integrate the passage of the Gaussian packet across the threshold during the delay Δ to estimate the mean number of spurious spikes, which we define as λ:

$$\lambda = N \int_{\theta-\delta/\tau}^{\theta} \frac{1}{\sqrt{2\pi}\sigma_{OU}} e^{-\frac{z^2}{2\sigma_{OU}^2}} dz. \tag{S85}$$

To understand this expression, note that between the time $t_{thresh,1}$ to time $t_{thresh,1} + \Delta$, the mean of the membrane potential distribution is shifting up at a rate N/τ. So over this duration of length $\Delta = \delta/N$ the mean shifts up by δ/τ. Thus the tail above threshold also shifts up. This integral reflects an integral over the additional probability mass of the membrane potential distribution that is above threshold between the time of the first spike $t_{thresh,1}$ and the time $t_{thresh,1} + \Delta$ of the first inhibition to all neurons. Multiplying this by N then yields an estimate for the mean number of spurious spikes λ. This simple approximation of Eq. (S83) to Eq. (S85) for the mean number of spurious spikes λ yields a good agreement with numerical simulations (Figure C) when the distribution of membrane potentials is actually drawn from a Gaussian. We note though that the approximation error consistently corresponds to a slight overestimate at finite N; this appears to be a finite size effect as this error diminishes as N becomes large, as demonstrated in (Figure C).

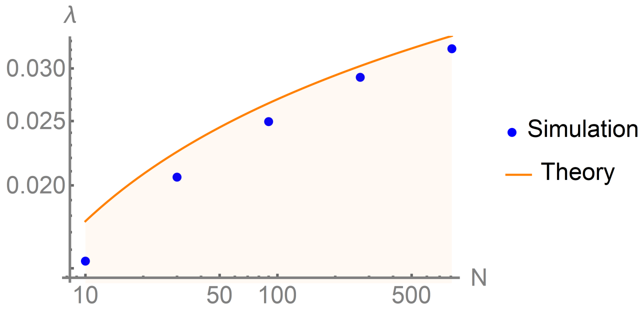

Figure C: The mean number of spurious spikes λ from Monte Carlo simulations is upper-bounded by Eq. (S85). In these simulations, we sample the number of spurious spikes 2×10^5 times, using the following procedure for each sample. First, we take N draws from a Gaussian of width $\sigma_{OU} = 1$ and mean zero, with each draw representing the value of a membrane potential relative to the mean membrane potential $\bar{V}$. Second, we take the maximum of these draws to represent the top neuron's membrane potential; we call this θ. Third, we count how many membrane potentials lie between θ and $\theta - \delta$ (we choose $\delta = \tau \times 10^{-2}$ for this figure), and record the count as the number of spurious spikes. Finally, we average over the 2×10^5 samples obtained using this procedure to compute the mean number of spurious spikes λ (blue dots). We also compute λ using Eq. (S83) to Eq. (S85) with the same parameters, $\sigma_{OU} = 1$ and $\delta = \tau \times 10^{-2}$ (orange curve).

Now importantly, we recall the detail that the $O(\Delta)$ time-difference between the top-neuron's self-reset and the inhibition of the rest of the population slightly stretches the upper tail of the membrane potential packet due to the leak term $-\lambda_V V_i(t)$ through the head start effect. Thus the actual tail of the membrane potential packet is slightly thinner than the Gaussian tail we used in our calculation for λ. However, we note that a thinner tail would facilitate even fewer spurious spikes, because the membrane potentials are more spread out. Thus our calculation for λ is a further overestimate, when this effect is taken into account. Also importantly, we have neglected variations due to the noise term $\sqrt{\tau}\sigma\eta_i(t)$ in the membrane potentials that may cause extra neurons to cross threshold and spuriously spike—but on the timescale of the delay Δ, these diffusive fluctuations have a standard deviation of $O(\sigma\sqrt{\Delta}) = O(\sigma\sqrt{\delta/N})$, which is much smaller than the mean membrane potential increase due to the driving current during the delay, which is $O(\Delta N) = O(\delta)$. Thus, these diffusive fluctuations can be safely neglected in computing the mean number of spurious spikes.

Importantly, the mean number of spurious spikes λ is small for high-performing networks. While we have computed an estimate of the mean here, we will further make the approximation that the distribution of the number of spurious spikes is Poisson. In the limit of small λ, where large numbers of spurious spikes are extremely rare, the detailed distribution of spurious spikes is not expected

to have a large impact on the readout error, and so a Poisson approximation with the same mean should suffice to approximately calculate the readout error. We will confirm this expectation below.

A.4.2 Mean readout error

With this knowledge of the distribution of spurious spikes in an event, let us compute $\sigma_{readout}$ by generalizing the calculation in the previous section. In the previous section, we focused on the case where $\lambda_V \ll 1$ for ease of analysis, and found empirically that this case provided an upper-bound for $\sigma_{readout}$ for the case of larger λ_V. Thus here, we will again start by analyzing the $\lambda_V \ll 1$ case, with the expectation that this corresponds to an upper-bound. Now, the primary difference between the dynamics of the network in the previous section with $\Delta = 0$, and this section with $\Delta > 0$, is that we have $1 + l$-spike events, where $l \geq 0$ as opposed to only 1-spike events. So to generalize our calculations for the variation of the readout at a particular time in the previous section, let us define here the value of the readout at a time t_{mid} which is $l\tau/N + \tau/2N$ after the start of an event, to be $\xi := \hat{x}(t_{mid})$. Since we are considering the case of high network performance, i.e. $\lambda \ll 1$, by far the most prevalent type of event is 1-spike events, followed by 2-spike events. Thus our calculation for the standard deviation σ_ξ of the random variable ξ in the previous section carries through to zeroth order in λ. But remarkably, one can also repeat the calculation for σ_ξ by imagining a readout consisting of only 2-spike events, and one recovers the same result, $\sigma_\xi = \sigma/\sqrt{2}N$. (Intuitively, the sums of spike-time variation $\sum_{n=1}^{k} \gamma_n$ in the calculation now contain half as many terms, but each γ_n has twice the variance because 2-spike events have twice the duration of 1-spike events and integrate twice as much noise $\sqrt{\tau}\sigma\eta_i(t)$.) Thus, aside from additional variance from the random interspersing of 2-spike events among 1-spike events, σ_ξ is still $\sigma/\sqrt{2}N$. Notably, we have seen in the soft-threshold model that such an additional variance is higher-order in $1/N$. However, in the LIF model, we also have the possibility that the membrane potential packet retains a leaky memory of its history, thus 2-spike events may tend to occur in *runs* of some typical length χ (a correlation length) as opposed to the single, randomly interspersed 2-spike events that we considered in the soft-threshold model. In the LIF model, a 2-spike event is more likely to follow a preceding 2-spike event than a preceding 1-spike event, because in the preceding 2-spike event, a second-to-top neuron is already close to threshold, ready to create a spurious spike with relatively high probability. To understand the magnitude of the additional variance contributed to σ_ξ^2 due to 2-spike event runs of length χ, one can repeat the calculation for Eq. (S45) with 2-spike event run replacements instead of individual 2-spike event replacements, and one finds that the additional variance to ξ scales as χ/N^3. So as long as χ scales $< O(N)$, we can still safely neglect this additional variance contribution, and our result of $\sigma_\xi = \sigma/\sqrt{2}N$ still holds to leading order in N.

Equipped with the variance σ_ξ^2 of the readout at time t_{mid}, the next step toward computing $\sigma_{readout}$ is computing the integrated squared deviation s_i

for an event, because $\sigma_{readout}$ is simply a sum of the s_i (Eq. (S20)). We can categorize events by spike-number just like we did in the soft-threshold model, so that we can use the simplified expression for $\sigma_{readout}$, Eq. (S30), that expresses the sum over all s_i as a weighted sum of expectation values of $s_i(l)$, where $s_i(l)$ is defined as s_i conditioned on the event containing $l+1$-spikes. To compute an instance of $s_i(l)$ then, we can take our expression for s_i in the previous section (Eq. (S75)) and modify it to include the fact that the event $s_i(l)$ contains l spurious spikes. We obtain the equation below, and explain the modifications we made in turn.

$$s_i(l) = \int_0^{t^i_{fp}(l)} \left[\left(\xi_i + \frac{1}{2N} + \frac{l}{N} - \frac{1}{\tau}t' \right) - \langle \hat{x}(t) \rangle_t \right]^2 dt'. \tag{S86}$$

First, we have introduced the random variable $t^i_{fp}(l)$, which is the duration of an $l+1$ spike event. Since the membrane potentials of an $l+1$-spike event must recover to threshold from a decrement of $l+1$, the moments of $t^i_{fp}(l)$ are given by the first-passage time distribution of a particle undergoing Brownian motion (this is using our assumption of small λ_V) with mean $(l+1)\tau/N$ and standard deviation that is $O(1/N^{3/2})$, which we can neglect from our calculations as we did in the previous section. Second, we have added the term $\frac{l}{N}$ to denote the increment in the readout near the beginning of the event due to the spurious spikes that occur within a time Δ from the beginning of the event. Note that we have collapsed the spike-times of the l spurious spikes over a time interval of size Δ, to a point time-interval at the beginning of the event. To understand why this approximation accounts for the leading-order effect of delays, recall that the time $\Delta \ll \tau/N$, and thus the contribution to the integral in Eq. (S86) from the $O(\Delta)$ spike-time variability of the l spurious spikes is much less than the contribution of the $\frac{l}{N}$ term, which is integrated over the entire $O(\tau/N)$ duration of the event. Thus, collapsing the spike-times serves to capture the leading-order effect of the delay Δ—that of the $\frac{l}{N}$ term.

Now, to evaluate Eq. (S86), we still need to compute the mean readout $\langle \hat{x}(t) \rangle_t$. But instead of going through another calculation to evaluate $\langle \hat{x}(t) \rangle_t$, we note that we are already describing an upper-bound for $\sigma_{readout}$ because the result from the previous section (Eq. (S82)) is an upper-bound for general λ_V, and our estimate for the number of spurious spikes λ (Eq. (S85)) is also an overestimate. Thus here too, we have the freedom to make an overestimate in our calculation of $s_i(l)$, and the usage of such an $s_i(l)$ will ultimately result in an upper-bound for $\sigma_{readout}$. We take advantage of this freedom to avoid the additional complexity of calculating $\langle \hat{x}(t) \rangle_t$, and instead substitute the result $\langle \hat{x}(t) \rangle_t = \mathbb{E}(\xi)$ from the case of zero delays (Eq. (S78)) into Eq. (S86). Clearly, $\langle \hat{x}(t) \rangle_t$ may deviate from this result because in the case of delay $\Delta > 0$, there are $1 + l$-spike events to consider, and not just 1-spike events. But crucially, the actual value of $\langle \hat{x}(t) \rangle_t$ minimizes the mean squared deviation $\sigma_{readout}$ because of a general property of computations for mean-squared-error (Eq. (S17)). Namely, calculating deviations relative to the mean of a random variable minimizes mean-squared error. Thus any value we substitute for $\langle \hat{x}(t) \rangle_t$ can only

yield the exact value or overestimate for the expectation of $s_i(l)$, $\langle s_i(l)\rangle_i$, which is the quantity we will use to compute $\sigma_{readout}$ in Eq. (S30). Therefore, let us implement this substitution, keeping in mind that this provides an overestimate. Eq. (S86) becomes

$$s_i(l) = \int_0^{t^i_{fp}(l)} \left[\left(\xi_i + \frac{1}{2N} + \frac{l}{N} - \frac{1}{\tau}t'\right) - \mathbb{E}(\xi)\right]^2 dt' \tag{S87}$$

$$= \int_0^{t^i_{fp}(l)} \left[\sigma_\xi y_i + \frac{1}{2N} + \frac{l}{N} - \frac{1}{\tau}t'\right]^2 dt' \tag{S88}$$

where we have cancelled $\mathbb{E}(\xi)$ in the second line and introduced the unit Gaussian random variable y_i to express the variation in ξ_i. Taking the expectation over the $s_i(l)$, we then obtain

$$\langle s_i(l)\rangle_i = \frac{1}{N^2}\frac{1}{12}(l+1)(1+2l+4l^2+6\sigma^2) \tag{S89}$$

Then, to compute $\sigma_{readout}$, we can substitute Eq. (S89) into Eq. (S30):

$$\sigma^2_{readout} = \frac{\sum_{l=0}^{\infty} p(l)\langle s_i(l)\rangle_i}{\sum_{l=0}^{\infty} p(l)(l+1)\tau/N} \tag{S90}$$

$$= \frac{1}{N^2}\left(\frac{\sigma^2}{2} + \frac{1+13\lambda+18\lambda^2+4\lambda^3}{12(1+\lambda)}\right) \tag{S91}$$

$$= \frac{1}{N^2}\left(\frac{1}{12} + \frac{\sigma^2}{2} + \lambda + O(\lambda^2)\right) \tag{S92}$$

where $p(l)$ is a Poisson distribution as defined in Eq. (S24), but with λ from Eq. (S85), and we recall that the denominator represents the mean duration of an event. Now, recalling that we made several overestimates during our derivation—the small λ_V limit corresponds to an upper-bound for $\sigma_{readout}$ for general λ_V, our estimate for λ was an overestimate (Eq. (S85)), and our choice to compute deviations relative to the $\mathbb{E}(\xi)$ instead of the mean readout was an overestimate—and that we made the central limit theorem approximation in the computation of σ_ξ, this expression for $\sigma_{readout}$ is in fact an approximate upper-bound. So we write

$$\sigma^2_{readout} \lesssim \frac{1}{N^2}\left(\frac{1}{12} + \frac{\sigma^2}{2} + \lambda\right). \tag{S93}$$

where we have also dropped high-order terms in λ because we are interested in networks with high performance ($\lambda \ll 1$).

Importantly, Eq. (S91) can be evaluated numerically (numerically evaluating Eq. (S85)) and compared against simulation (Figure 4). Furthermore, we can numerically compute the minimal readout standard deviation, $\sigma^*_{readout}$, and the associated optimal noise level, σ^* (Figure 4). Finally, we numerically differentiate $\sigma^*_{readout}$ and σ^* with respect to the delay δ and observe that $\sigma^*_{readout}$

and σ^* grow as

$$\sigma^*_{readout} \sim (\delta/\tau)^{2/3} \qquad \text{and} \tag{S94}$$

$$\sigma^* \sim (\delta/\tau)^{1/3} \tag{S95}$$

for $\delta \ll \tau$, which matches the behavior of the soft-threshold model, Eq. (S39) and Eq. (S38) (Figure D).

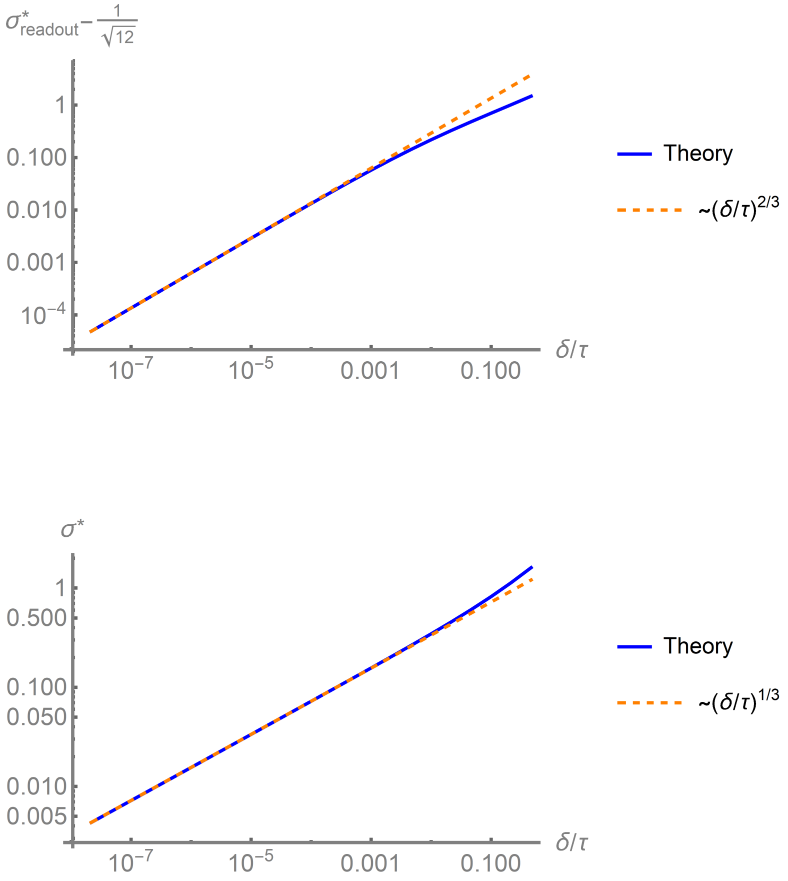

Figure D: Numerically minimizing Eq. (S91) with respect to σ, we find that $\sigma^*_{readout}$ and σ^* (top/bottom, solid blue curve) grow with small delay $\delta \ll \tau$ as $\sim (\delta/\tau)^{2/3}$ and $\sim (\delta/\tau)^{1/3}$, respectively (top/bottom, dashed orange line). Note that this limiting behavior matches that of the soft-threshold model. Noise enters the soft-threshold model as the standard deviation of the first-spike time, $\frac{1}{N\rho}$. And in the soft-threshold model, $\lambda^* \sim (\delta/\tau)^{2/3}$ implies that $\frac{1}{\rho*} = \frac{\delta}{\lambda^*} \sim (\delta/\tau)^{1/3}$, which matches the $\sigma^* \sim (\delta/\tau)^{1/3}$ that we have here for the LIF model.

B Simulation details

We use $\tau = 1$ in all simulations.

B.1 Soft-threshold model

In Figure 2b, our simulations take advantage of the small delay Δ ($\Delta = \delta/N$, where $\delta \ll \tau$) and large N limit of the soft-threshold model to efficiently compute spike-times t_k in Eq. (S6) and empirically estimate $\sigma_{readout}$. We first consider when all the neurons cross threshold together, and the neurons spike with total probability rate $N\rho$. The first-spike time is an exponential random variable with mean $1/N\rho$, and so in our simulations, we simply draw the first-spike time from this exponential distribution. Next, we consider the time after the first spike, during the delay Δ. The other $N - 1 \approx N$ neurons continue to spike probabilistically, with each neuron's next-spike time exponentially distributed with mean $1/\rho$, in the absence of inhibition. Here we draw N next-spike times from this exponential distribution. Of course, inhibition arrives after a time Δ, and so the only neurons that spike are the ones with next-spike time less than Δ; we take these as the spuriously spiking neurons, and their spike times are given by the first-spike time, plus the next-spike time for each neuron. After the inhibition arrives from the first-spike neuron, the entire population is subthreshold, and the population returns to threshold a time $(1 + l)\tau/N$ after the first spike, where l is the number of spurious spikes. At this point, we repeat our procedure, starting by drawing a new first-spike time, and so on. This yields a list of spike-times, which we insert into Eq. (S6). We use $N = 32$ neurons, run the simulation for 3×10^4 first-spikes, and sample 1.5×10^4 random times in $\hat{x}(t)$ to empirically calculate $\sigma_{readout}$. We sample from within the last 2×10^4 spikes in the simulation, to ensure that the readout has reached the steady state.

This simulation protocol has the advantage that it avoids traditional Euler-timestep discretized time, which allows us to easily examine small delays. However, notably, this protocol does not consider the possibility that the first spike takes so long to occur that the population travels so far above threshold and the first spike is insufficient to return the population to subthreshold. Importantly, for small δ, ρ is large, and the mean first-spike time is small. Furthermore, very large first-spike times are exponentially suppressed; thus the simulation is accurate for small δ.

B.2 LIF model

In Figures 3 and 4, we perform Euler time-step simulations of Eq. (S1) with $dt = 0.0001$, and we use the second half of the simulation when computing $\sigma_{readout}$.

For Figure 3a, we use $N = 32$, $\sigma = 0.1$, and 1562500 time-steps in each simulation (each dot). For Figure 3c, we use $N = 64$ and 781250 time-steps.

For Figure 4d, we use $\sigma = 0.001, 0.003, 0.01, 0.03, 0.1, 0.3, 1., 3., 10.,$ or 30., and 781250 time-steps for each simulation. In Figure 4e, we take the minimum over the $\sigma_{readout}$s from Figure 4d, for each delay value δ, to compute $\sigma^*_{readout}$ and σ^*.

B.3 Simulation code

We include here our Euler time-step simulation code used for the LIF model. We have described the other, simpler computations supporting this work directly in the text.

```
# Julia 1.3.1
using LinearAlgebra

# Spiking neural network simulation
function snn_sim(;
    D = 1,                  # Dimension of x(t), NOTE D=1 for this work
    N = 32,                 # Number of neurons
    lambda_V = 0.1,         # Membrane leak
    sigma_V0 = 0.,          # Standard deviation of membrane potential
                            # initialization
    n_steps = 1562500,      # Number of simulation time steps
    n_step_save = 5000,     # Number of steps between each snapshot save of
                            # simulation state
    n_step_delay = 30,      # Length of axonal propagation delay in number of
                            # time steps
    hard_spike = true,      # true => hard threshold, false => soft threshold
    dt = .0001,             # Time step size
    W_input = Nothing,      # Input weight connectivity matrix
    membrane_noise = 1.,    # Noise level sigma
    thres = Nothing,        # Neuron thresholds
    prob_fire_in_dt = 1.,   # Probability of spiking during time step dt (for
                            # soft threshold)
    omega_f = Nothing,      # Connectivity weight matrix
    read_out = Nothing,     # Readout weights
    A = Nothing,            # Emulated D-dimensional dynamical system
    only_one_spike_per_dt = false # true => only allow 1 spike per time step
                                  # (for zero delay), false => multiple
                                  # neurons can spike in a single time step
    )

    # Example values
    if W_input == Nothing
        W_input = N * ones(N,D)
    end
    if omega_f == Nothing
        omega_f = ones(N, N)
    end
    if read_out == Nothing
```

```
    read_out = 1.0 / N * ones(1, N)
end
if thres == Nothing
    thres = 0.5 * ones(N)
end

# Setup for emulating x(t) as a low-pass filter of constant 1-D input c(t) = 1
if A == Nothing
    A = -1. * I
end
c = ones(n_steps,1)

# Initialize simulation parameters
V0 = sigma_V0 * randn(N) # Membrane potentials
r0 = zeros(N) # Firing rates
x0 = [0.] # x(t)

V = copy(V0)
x = copy(x0)
r = copy(r0)

# Storage for simulation history
history_length = trunc(Int, n_steps/n_step_save)
V_history = zeros(history_length, N)
x_history = zeros(history_length,D)
r_history = zeros(history_length,N)
x_hat_history = zeros(history_length,D)

# Initialize readout error metrics
x_hat_squared_sum = 0.
x_hat_sum = 0.
error_calc_start = trunc(Int, n_steps/2) # Burn-in period

# Delayed spike delivery queue and spike time list
spike_queue = []
spike_times = []

# Euler time-step simulation
for step in 1:n_steps

    V_dot = -lambda_V * V + W_input * c[step,:]

    if membrane_noise != 0.
        V_dot += membrane_noise/sqrt(dt) * randn(N)
    end
```

```
V += V_dot * dt

# Check for neurons crossing threshold
for n in 1:N
    if V[n] > thres[n]
        if hard_spike || rand() <= prob_fire_in_dt
            index_and_delivery = (n, step + n_step_delay)
            push!(spike_queue, index_and_delivery)
            push!(spike_times, index_and_delivery)
            V[n] -= omega_f[n,n] # Immediate self-reset

            if only_one_spike_per_dt
                break
            end
        end
    end
end

# Deliver delayed spikes
while length(spike_queue) > 0 && step >= spike_queue[1][2]
    spike_index = splice!(spike_queue,1)[1]
    V -= omega_f[:,spike_index]
    V[spike_index] += omega_f[spike_index, spike_index] # Undo extra
                                                         # self-reset from
                                                         # previous line
    r[spike_index] += 1.
end

# Update state of D-dimensional dynamical system
x_dot = A * x + c[step,:]
x += x_dot * dt

# Update firing rates and output
r_dot = -r
r += r_dot * dt

x_hat = read_out * r

# Compute error starting after burn-in period
if step > error_calc_start
    x_hat_squared_sum += norm(x_hat) ^ 2
    x_hat_sum += norm(x_hat)
end

# Log simulation parameters
if step % n_step_save == 0
```

```
            history_index = trunc(Int, step/n_step_save)
            V_history[history_index, :] = V
            r_history[history_index, :] = r
            x_history[history_index, :] = x
            x_hat_history[history_index, :] = x_hat
        end

    end

    sigma_readout2 = (x_hat_squared_sum / (n_steps - error_calc_start) -
                     (x_hat_sum / (n_steps - error_calc_start))^2 )

    return Dict(
    :N => N,
    :D => D,
    :V_history => V_history,
    :r_history => r_history,
    :x_history => x_history,
    :x_hat_history => x_hat_history,
    :spike_times => spike_times,
    :sigma_readout2 => sigma_readout2,
    )

end
```

References

[1] George E Uhlenbeck and Leonard S Ornstein. On the theory of the brownian motion. *Physical review*, 36(5):823, 1930.

[2] Henry C Tuckwell. *Introduction to theoretical neurobiology: volume 2, nonlinear and stochastic theories*, volume 8. Cambridge University Press, 1988.
